# Climatic and ecological responses to Bennu-type asteroid collisions

**DOI:** 10.1126/sciadv.adq5399

**Published:** 2025-02-05

**Authors:** Lan Dai, Axel Timmermann

**Affiliations:** ^1^ Center for Climate Physics, Institute for Basic Science (IBS), Busan 46241, Republic of Korea.; ^2^ Pusan National University, Busan 46241, Republic of Korea.

## Abstract

There is an estimated chance of 0.037% that asteroid Bennu will collide with Earth in 2182 CE. The potential collision of such medium-sized asteroids can inject massive amounts of dust into the atmosphere, with unknown consequences for terrestrial and marine ecosystems. Here, we use the coupled high-top Community Earth System Model Version 2 with interactive chemistry to investigate how medium-sized asteroid strikes would affect climate, vegetation, and marine productivity. Our simulations, which inject up to 400 million tons of dust into the stratosphere, show marked disruptions in climate, atmospheric chemistry, and global photosynthesis. Global mean temperatures are projected to drop by 4°C, and global precipitation decreases by 15% in our simulations. The largest relative reductions in global terrestrial and marine net primary productivity reach 36 and 25%, respectively. Depending on the iron amount of the asteroid and the subsequent marine dust deposition, large diatom blooms occur in iron-limited regions such as the Southern Ocean and the eastern equatorial Pacific.

## Teaser

Massive disruptions in climate and global ecosystems are simulated for Bennu-type asteroid strikes.

## INTRODUCTION

Several near-Earth asteroids (NEAs) in the solar system pose non-negligible threats to the habitability of our planet (*1*, *2*). As of September 2024, more than 35,800 NEAs have been found, and about 4925 of them are medium-sized asteroids with diameters between 300 m and 1 km (*3*). Asteroids with diameters of about 1 km are statistically estimated to strike Earth every 600,000 to 700,000 years (*4*). Although the impact probability is extremely low, it cannot be completely ruled out that medium-sized asteroids will collide with Earth within the next two centuries. One of the known, potentially most hazardous asteroids, Bennu, with a diameter of ~500 m has been predicted to strike our planet in September 2182 with a 1-in-2700 chance (*5*).

Depending on the collision parameters, an impact between medium-sized asteroid and Earth could cause regional to large-scale devastation (*6*, *7*). Beyond immediate effects such as thermal radiation, earthquakes, and tsunamis, asteroid impacts would have long-lasting climatic effects by emitting large quantities of aerosols and gases into the atmosphere (*8*). Previous studies on the much larger Chicxulub 10-km asteroid impact ~66 million years ago indicate that three groups of climate-active ejecta, consisting of dust, soot, and sulfur, contributed to the global “impact winter” and very likely triggered the dinosaur mass extinction at the Cretaceous/Paleogene (K/Pg) boundary (*9*–*15*). Soot particles from global wildfires and sulfate aerosols from target evaporites probably played more important roles in the prolonged “impact winter” due to their longer stratospheric residence times (*10*–*14*). By contrast, other studies have pointed out that more abundant dust particles played a large part in driving the global darkness and prolonged collapse of plant photosynthesis (*9*, *15*).

Less attention has been paid to the effects of medium-sized asteroid collisions, which are far more frequent than the “planet killer” asteroids but yet still can have marked global consequences (*16*). Recent work investigated ozone perturbations by water injections from impacts with 500-m and 1-km sized asteroids in the northern subtropical Pacific Ocean (*17*). Another extended study simulated the climatic effects of submicron dust particles and soot from a 1-km-sized asteroid impact on land (*18*). However, the responses of global climate and terrestrial and marine ecosystems to different dust injections from medium-sized asteroid impacts on land remain uncertain. In this study, we use the state-of-the-art high-top coupled Earth system model [Community Earth System Model Version 2 (CESM2); (*19*)] at 0.9° × 1.25° horizontal resolution with fully interactive atmospheric chemistry [Whole Atmosphere Community Climate Model Version 6 (WACCM6); (*20*)] and land and ocean biogeochemistry to investigate the effects of dust injections from potential medium-sized asteroid impacts on our Earth system. We perform a set of idealized injection experiments to quantify the physical, chemical, and biological responses of the climate system and terrestrial and marine ecosystems to different dust forcings after the impact (see Materials and Methods). Our initial dust perturbation is applied with an idealized initial horizontal and vertical distribution (figs. S1 and S2A).

## RESULTS

### Evolution of dust particles in the atmosphere

**Fig. 1.**
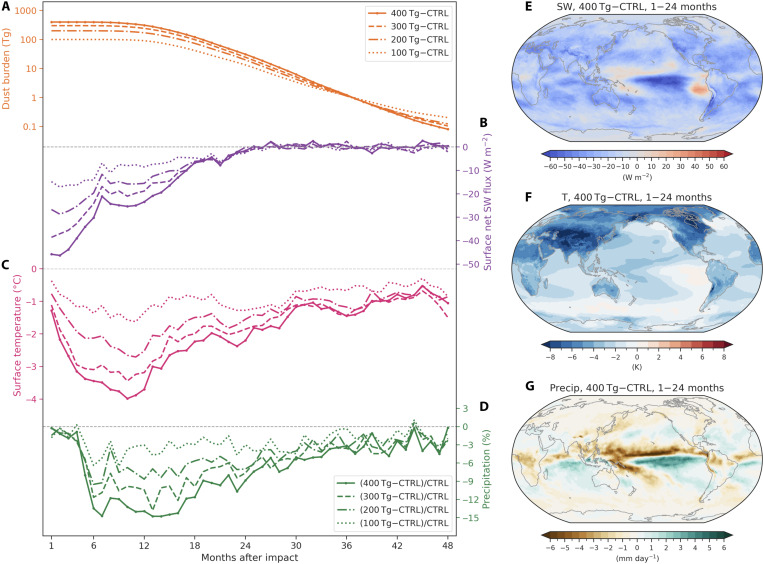
Global responses of the climate system to dust perturbations after a Bennu-type impact under four dust scenarios. Time series of globally and monthly averaged absolute changes in (**A**) total column dust burden (note *y* axis is shown in log scale), (**B**) surface net shortwave flux, (**C**) surface temperature, and percentage changes in (**D**) precipitation rate between four dust simulations and the control simulation over 48 months. Dust injections of 100, 200, 300, and 400 Tg in January are shown as dotted, dash-dotted, dashed, and solid lines, respectively. The light black dashed lines represent zero values. Spatial patterns of (**E**) surface net shortwave flux, (**F**) surface temperature, and (**G**) precipitation anomalies averaged over the first 2 years after the impact in the 400 Tg of dust simulation. All the anomalies in this study are calculated by subtracting the monthly data over 48 months in the control simulation.

The climate effects of impact-dust aerosols depend mostly on the total dust loading and its evolution in the atmosphere. After initial injections near the stratopause at 50 km (about 1 hPa; see Materials and Methods for more details), nearly 90% of total dust nanoparticles stay in the atmosphere for almost 1 year in four initial dust perturbation scenarios of 100, 200, 300, and 400 Tg of dust (Fig. 1A and fig. S3A). Large amounts of dust particles settle out of the atmosphere in the second year, with merely less than 10% (~10 to 25 Tg) of total dust remaining airborne 2 years after the impact. Dust particles fall out of the atmosphere more quickly in the 400 Tg of dust scenario because higher concentration leads to rapid coagulation and larger particles. Our simulations suggest that dust particles have much longer atmospheric residence times than for the Chicxulub impact simulation with 2,000,000 Tg of dust forcing, which shows a very quick removal of 99% dust within 3 months (*13*). It implies that relative to the total forcing, small dust injections from medium-sized asteroid impacts may have longer-lasting influences on the climate system. Previous work on soot simulations also reveals that the atmospheric lifetime of soot is much longer in the smaller injection scenario (*11*). Aerosol coagulation plays an important role in accelerating the deposition rate, and a lack of this process may lead to an overestimation of aerosol lifetime in some models (*15*).

The spatial evolution of dust aerosols also plays an important role in affecting regional climate responses. Dust particles are initially injected over the western hemisphere across all latitudes in boreal winter (January) but quickly disperse to all longitudes around the globe due to strong zonal winds in the stratosphere (fig. S3B). Initially, the majority of dust particles are confined to the Northern Hemisphere (NH) surrounding the stratospheric polar vortex in boreal winter and are then transported to the Southern Hemisphere (SH) in austral winter by the Brewer-Dobson circulation (fig. S3C). Dust dispersal is strongly controlled by seasonal changes of the stratospheric circulation and the time of the aerosol injection. If initially injected in the boreal summer (June), then a large portion of dust is transported poleward and concentrated in the winter (southern) hemisphere for about 6 months after the impact (fig. S3D). The meridional asymmetry of dust distribution in the NH and SH can cause distinct regional climate responses during the first year.

### Physical responses of the climate system to dust perturbations

Changes in monthly averaged surface net solar flux, surface temperature, and total precipitation in four dust injection scenarios relative to the control simulation are summarized in Fig. 1 (B to G). The globally dispersed dust particles largely scatter incoming solar radiation, resulting in a substantial reduction in global mean surface shortwave radiation lasting for almost 2 years after the impact (Fig. 1B). Surface net shortwave flux anomalies in four dust scenarios (100, 200, 300, and 400 Tg) respond almost linearly to the total dust forcings. The largest reduction in global mean surface net shortwave flux amounts to about −46 W/m^2^ (~28%) relative to the control simulation, as the global visible optical depth reaches a maximum value of ~2 shortly after the 400 Tg of dust injection (fig. S4). In all the simulated dust scenarios, surface net shortwave flux recovers to the unperturbed control state with anomalies of less than ~1 W/m^2^ after about 24 months (Fig. 1B). The recovery process is relatively consistent with the dust lifetime in the atmosphere (Fig. 1A). Changes in surface net solar flux in the 400 Tg of dust case indicate notable regional variability (Fig. 1E). There are widespread reductions in shortwave radiation over the globe except for the tropical Pacific Ocean due to atmospheric circulation changes and a reduction in cloud cover (fig. S5A).

In addition to the direct radiative effect, dust particles also modify the cloud radiative effects through interactions with clouds. The global mean cloud cover exhibits large reductions between 300 and 100 hPa in the upper troposphere and slight increases in the lower and middle troposphere (fig. S5B). The anomalous vertical cloud dipole pattern is closely linked to surface cooling, decreased water vapor, and suppressed convection. The radiative forcing of reduced total clouds shows a weakening of the net cooling effects of clouds for more than 2 years after the impact, which eventually contributes to planetary warming (fig. S5, C and D). The net warming tendency is the result of a stronger reduction in negative shortwave effects as compared to the positive longwave effects (fig. S5D).

Our simulations show marked global surface cooling in response to all dust perturbation scenarios (Fig. 1C), attaining values of about −1.6°, −2.7°, −3.4°, and −4.0°C for dust injections of 100, 200, 300, and 400 Tg, respectively. The maximum cooling of 4.0°C in the 400 Tg of dust scenario is comparable with the volcanic cooling estimated for 2000 Tg of SO_2_ emission from the Toba eruption ~74,000 years ago (*21*). Surface temperature cools more over land especially in the NH than over ocean due to the slow response in the ocean, the increased heat capacity, and the evaporation-temperature feedback (Fig. 1F). Eurasia and North America experience the most rapid and strongest cooling since much of the dust is concentrated in the NH in boreal winter (fig. S3C). Anomalous warming over the eastern equatorial Pacific resembles a weak El Niño-like pattern at the end of first year, which is similar to the El Niño response in nuclear winter simulations (*22*). Global mean surface cooling persists for more than 4 years, much longer than the shortwave anomalies, with slow recovery to within −1°C of the normal state after 24 months (Fig. 1C).

Unlike the more gradual cooling response, global mean precipitation rate initially shows little response, but then after 5 months, we observe an abrupt decline (Fig. 1D). The nonlinearity can be explained by the fact that in the initial 4 months positive (tropical Indian Ocean, South Pacific, and tropical South Atlantic) and negative (tropical Pacific, southern Africa, eastern Australia, and South America) precipitation anomalies compensate each other over the tropics (fig. S6A). This precipitation pattern is generally consistent with the anomalous distribution of cloud cover and water vapor in the tropics, which is closely linked to distinct spatial responses of tropical sea surface cooling (fig. S6, B to D). The global mean atmospheric water content decreases simultaneously with surface temperatures in the first few months after the impact (Fig. 1C and fig. S7). However, the total cloud cover responds nonlinearly to temperature decrease, with fluctuant negative and positive anomalies in the initial 4 months (fig. S5C).

Thereafter, the overall Clausius Clapeyron effect from global cooling becomes dominant, weakening the global hydrological cycle by 5 to 15% from 5 to 30 months after the impact in the 400 Tg of dust scenario. The maximum reduction in global mean precipitation is about −0.46 mm/day (~15%) after 6 months, and the pattern is characterized by an equatorward shift of the Intertropical Convergence Zone (ITCZ) and the South Pacific Convergence Zone (SPCZ) and an increase of precipitation in the marine subtropics and massive drying elsewhere (Fig. 1, D and G). Previous studies have suggested that the ITCZ tends to shift away from the hemisphere with more aerosol forcing and stronger cooling (*23*). The ITCZ over the Pacific weakens with the zonal-mean precipitation maxima decreased by −4.32 mm/day (~43%) around 7° north of the equator (fig. S8A). The southward shift of the ITCZ contributes to precipitation increases between 10°S to 2°N with the maxima of 2.94 mm/day near 4°S. The annual-mean position of the ITCZ in the South Asian monsoon sector migrates from 3°N to 4°S, with changes in precipitation maxima from 8.14 to 7.93 mm/day (fig. S8B). Large parts of land areas experience a substantial reduction in precipitation of about 30 to 60%, especially over southern Africa, Eurasia, North America, and northern South America (fig. S9A). There are also widespread decreases in evaporation over land areas with reduced precipitation (fig. S9B). The pattern of precipitation minus evaporation shows strong moisture deficit and substantial drying over southern Africa, northern South America, and parts of South Asia (fig. S9C).

### Chemical responses of stratospheric ozone to dust perturbations

**Fig. 2.**
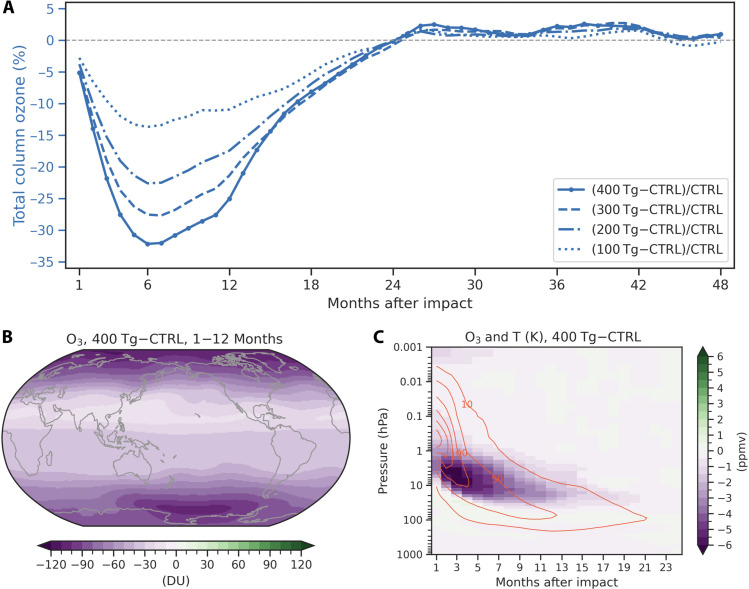
Stratospheric ozone response to dust perturbations after a Bennu-type impact. (**A**) Time series of globally and monthly averaged percentage changes in total column ozone relative to the control simulation over 48 months for four dust scenarios. Dust injections of 100, 200, 300, and 400 Tg in January are shown as dotted, dash-dotted, dashed, and solid lines, respectively. The light black dashed lines represent zero values. (**B**) Spatial pattern of total column ozone anomalies averaged over 12 months after the impact in the 400 Tg of dust simulation. (**C**) Vertical distribution of ozone concentration (shading) and temperature (contours) anomalies over 24 months.

Massive dust injections into the stratosphere play a vital role in the stratospheric ozone chemistry. Our simulations exhibit a substantial decline in the total column ozone for about 24 months after the impact, with the global mean ozone dropping below 189 Dobson units (DU) in boreal summer and the maximum ozone loss reaching −90 DU (~32%) relative to the control case (Fig. 2A). The ozone loss is more pronounced over the mid-to-high latitudes in both hemispheres than in the tropics (Fig. 2B). Substantial ozone depletion of up to −6 parts per million by volume extends from 1 to 30 hPa in the stratosphere, which is closely linked to the vertical distribution of dust aerosols (Fig. 2C and fig. S3A). On the one hand, dust absorbs the incoming shortwave radiation and reduces the radiation needed for ozone production (*24*). On the other hand, owing to the local radiative heating generated by dust-induced light absorption, stratospheric warming exceeds up to 100°C between 1 to 10 hPa in the first 4 months and extends downward to below 200 hPa in the following 24 months (contours in Fig. 2C). High temperatures in the stratosphere lead to ozone destruction by accelerating the catalytic reaction cycles of odd oxygen and odd nitrogen (*25*). Stratospheric ozone depletion has a harmful effect on the ecosystems and human health due to increased ultraviolet (UV) radiation to the surface (*26*). The explicit calculation of surface UV is not supported in our model configuration. Nevertheless, when comparing ozone concentration with surface solar flux, we propose that it is possible that the increased ozone loss compensates a portion of UV radiation to the surface, contributing to relatively less reduction in surface solar flux in the summertime of the first year (fig. S10). Another study about the dinosaur-killing asteroid simulations suggests marked increases in surface UV radiation after sufficient deposition of soot aerosols (*11*). However, massive amounts of dust aerosols in the stratosphere largely scatter the solar radiation and therefore prevent much of the UV radiation from reaching the surface, which could partially offset the risks of extensive surface UV exposure caused by ozone depletion. This hypothesis needs to be explored more in detail in future studies using a fully interactive UV radiation calculation scheme.

### Disruptions of photosynthesis in terrestrial and marine ecosystems

**Fig. 3.**
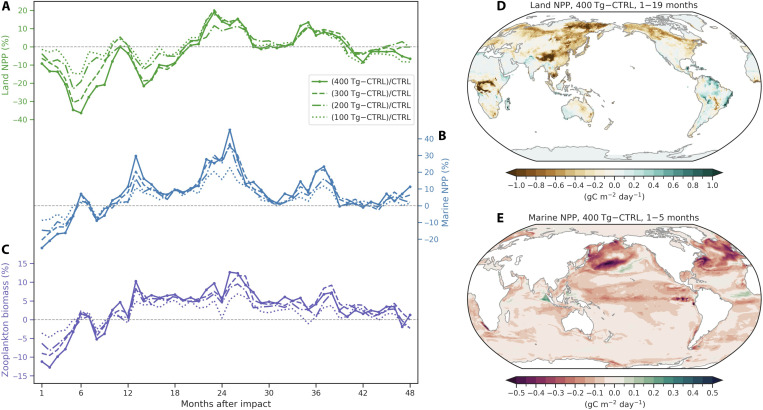
Simulated changes in terrestrial and marine NPP after a Bennu-type impact under four dust scenarios. Time series of global percentage changes in (**A**) land NPP, (**B**) marine NPP, and (**C**) zooplankton biomass relative to the control run over 48 months. Dust injections of 100, 200, 300, and 400 Tg in January are shown as dotted, dash-dotted, dashed, and solid lines, respectively. The light black dashed lines represent zero values. Spatial patterns of (**D**) land NPP anomalies averaged from 1 to 19 months and (**E**) marine NPP anomalies averaged from 1 to 5 months after the impact in the 400 Tg of dust simulation.

**Fig. 4.**
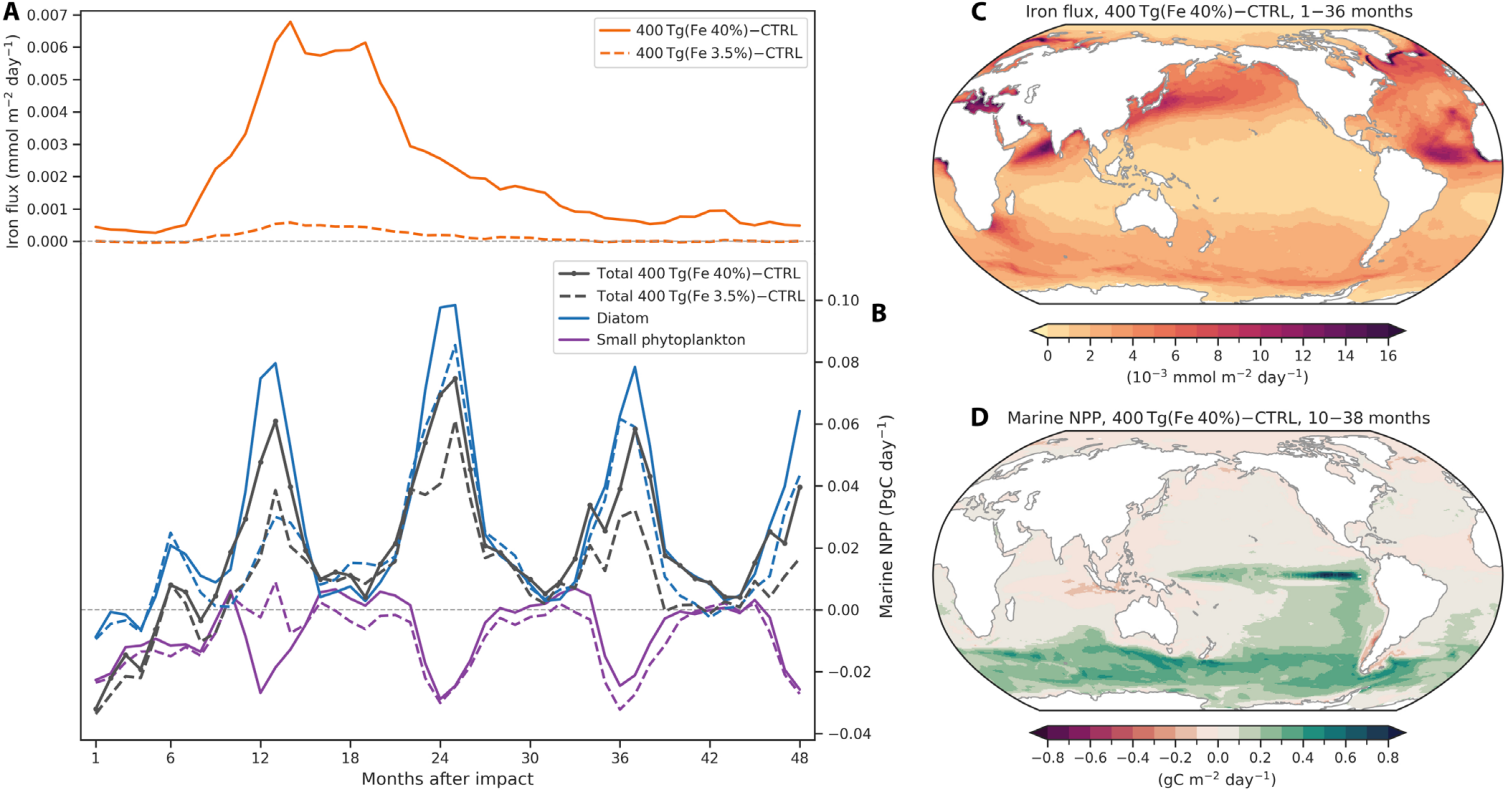
Changes in iron nutrient and marine NPP after a Bennu-type impact in two 400 Tg of dust simulations. Time series of monthly absolute changes in (**A**) globally averaged atmospheric iron flux into the ocean and (**B**) globally integrated anomalies of marine NPP relative to the control run over 48 months. Two 400 Tg of dust injection simulations with 3.5 and 40% iron contents are shown as dashed and solid lines, respectively. The light black dashed lines represent zero values. Diazotroph NPP is not shown in the figure because it is relatively smaller compared to diatom and small phytoplankton. Spatial patterns of (**C**) atmospheric iron flux anomalies averaged from 1 to 36 months, and (**D**) marine NPP anomalies averaged from 10 to 38 months after the impact in the 400 Tg of dust simulation with 40% iron content.

The global “impact winter” characterized by reduced sunlight, cold temperature, and decreased precipitation creates unfavorable climate conditions for plant growth in terrestrial and marine ecosystems. Figure 3 shows simulated changes in terrestrial and marine net primary productivity (NPP) relative to the control simulation under a range of dust scenarios. Initially, global total land NPP declines by a maximum of −23.01 petagrams of carbon (PgC)/year (~36%) in the boreal summer following the impact (Fig. 3A), while the globally integrated marine NPP drops by −12.29 PgC/year (~25%) already in the first month (Fig. 3B). Globally integrated zooplankton biomass changes in line with marine phytoplankton, with a maximum decrease of −0.02 PgC (~13%) in the 400 Tg of dust case (Fig. 3C). The recovery processes of ecosystem productivity on land and in the ocean are largely different. There are persistent decreases in land NPP over tropical western Africa and the mid-to-high latitudes in the NH lasting for almost 19 months (Fig. 3D). Land NPP slightly increases over parts of South America, Northeast Africa, Northwest India, and Southwest Australia, owing to less cooling and more precipitation in these regions (Figs. 1, F and G, and 3D). Terrestrial ecosystem productivity recovers beyond the pre-impact level during months 22 to 27 and 34 to 39 in the austral spring and summer (Fig. 3A). The enhanced NPP mainly occurs in the SH, especially over eastern Australia and large parts of South America (fig. S11). Terrestrial NPP exhibits different spatial responses in each hemisphere, with substantial reductions in the NH and slight increases in the SH (fig. S12A). These hemispheric differences can be explained by the varied responses of several main climatic factors. The combined effects of reduced photosynthetically active radiation (PAR), cold temperature, and less precipitation limit vegetation growth in the NH and cause persistent NPP reductions for nearly 4 years (fig. S12, B to D). The SH experiences relatively less stress from reduced PAR and cold temperature compared to the NH (fig. S12, B and C). In addition, increased precipitation contributes to enhanced NPP in the SH during the austral spring and summer at the end of the second and third years (fig. S12D). However, strong reductions in marine NPP only persist for the first 5 months due to reduced sunlight over North Atlantic, Northwest Pacific, and tropical Pacific (Fig. 3E). Marine phytoplankton shows an early recovery and a quick rebound in austral summer at the end of the first year, which is due to the gradual increase in shortwave radiation (Fig. 1B) and the deposition of iron-rich dust in the ocean—both of which can boost regional productivity (Fig. 4).

### Diatom blooms due to iron-rich dust deposition and La Niña conditions

Upper ocean iron serves as a micronutrient and controls the phytoplankton primary productivity in high nutrient low chlorophyll (HNLC) regions, such as the Southern Ocean and the eastern equatorial Pacific (*27*, *28*). External inputs of bioavailable iron from atmospheric dust deposition therefore play an important role in phytoplankton growth in these HNLC regions (*29*). The globally averaged iron content of mineral dust in Earth’s upper crust is 3.5%, which is a typical value used in Earth system models (*30*, *31*). In our study, the impact-generated dust particles originate from rocks of asteroids and Earth’s surface. Some metal-rich asteroids have a much higher abundance of iron (*32*). Moreover, extraterrestrial dust has also been shown to be an important source of bioavailable iron to the upper ocean (*33*).

To investigate the effects of different iron content of impact-generated dust on marine ecosystems, we compare two simulations with same dust injections of 400 Tg but different iron contents of 3.5% (standard simulation) and 40%, respectively (see Materials and Methods). Global mean atmospheric iron flux anomalies into the ocean increase substantially from 0.07 mmol/m^2^ per year in the 3.5% iron case to 0.95 mmol/m^2^ per year in the 40% iron case averaged over 36 months after the impact (Fig. 4A). Starting from 10 to 48 months after the impact, we observe large diatom blooms and concomitant reductions in small phytoplankton from October to the following February (Fig. 4B). Iron-rich dust deposition provides large amounts of iron as a micronutrient to the ocean, which greatly promotes diatom growth in iron-limited regions (Fig. 4C). Diatoms, which dominate the total NPP, and small phytoplankton compete for nutrients over the Southern Ocean and the tropical Pacific (Fig. 4D and fig. S13).

The pronounced diatom blooms and small phytoplankton reductions in the Southern Ocean are highly correlated with enhanced iron nutrient from dust deposition (Fig. 4, C and D). The relative abundance of diatoms and small phytoplankton in the Southern Ocean is mainly constrained by iron and light availability due to high concentrations of macronutrients (*28*). Nutrient and light limitations on the growth rates of diatoms and small phytoplankton are calculated prognostically in the marine biogeochemical model (*34*). Iron limitation on diatom growth is more severe than on small phytoplankton in the Southern Ocean in the unperturbed control simulation (fig. S14). Small phytoplankton has lower half-saturation constants of nutrient uptake in our model and is more efficient at nutrient uptake under iron-limited conditions compared to the larger diatoms (*34*). Additional iron inputs from dust deposition alleviate the limitations on both diatom and small phytoplankton in the two dust simulations. Starting from 11 to 34 months, iron is no longer the most limiting nutrient under extremely high iron concentrations in the 40% iron content simulation (fig. S14). In addition to nutrient limitation, light availability also plays an important role in phytoplankton growth in the Southern Ocean. The light harvesting capacity of diatoms is higher than small phytoplankton in the model (*34*). Small phytoplankton becomes more strongly limited by light in austral spring and summer under iron-rich conditions in the two 400 Tg of dust simulations (fig. S15). Diatoms have a competitive advantage over small phytoplankton under nutrient-rich conditions in the Southern Ocean due to the relief of iron limitation and high light harvesting efficiency.

The responses of small phytoplankton production to different impactor-iron concentrations vary with time after the impact in two 400 Tg of dust simulations (Fig. 4B). The globally integrated small phytoplankton NPP shows strong negative anomalies during months 11–14 but slightly positive anomalies during months 16–40 in the iron-richer (40%) simulation compared to the default iron (3.5%) simulation. This difference is dominated by the changes in small phytoplankton production in the Southern Ocean (fig. S16). During the first main growing season in the Southern Ocean (months 11–14) after the impact, moderate iron fertilization promotes the growth rates of both diatoms and small phytoplankton in the 3.5% iron simulation (fig. S17). However, more abundant iron and relatively limited light stimulate the explosive diatom growth but largely inhibit the growth of small phytoplankton in the iron-richer (40%) simulation. As for the following two austral spring and summer seasons (months 23 to 26 and 35 to 38), iron concentrations in the two dust simulations are both sufficient to promote the exclusive growth of diatoms. Small phytoplankton growth is thereby largely suppressed with NPP attaining values of less than 0.003 PgC/day in the two dust simulations (fig. S17).

**Fig. 5.**
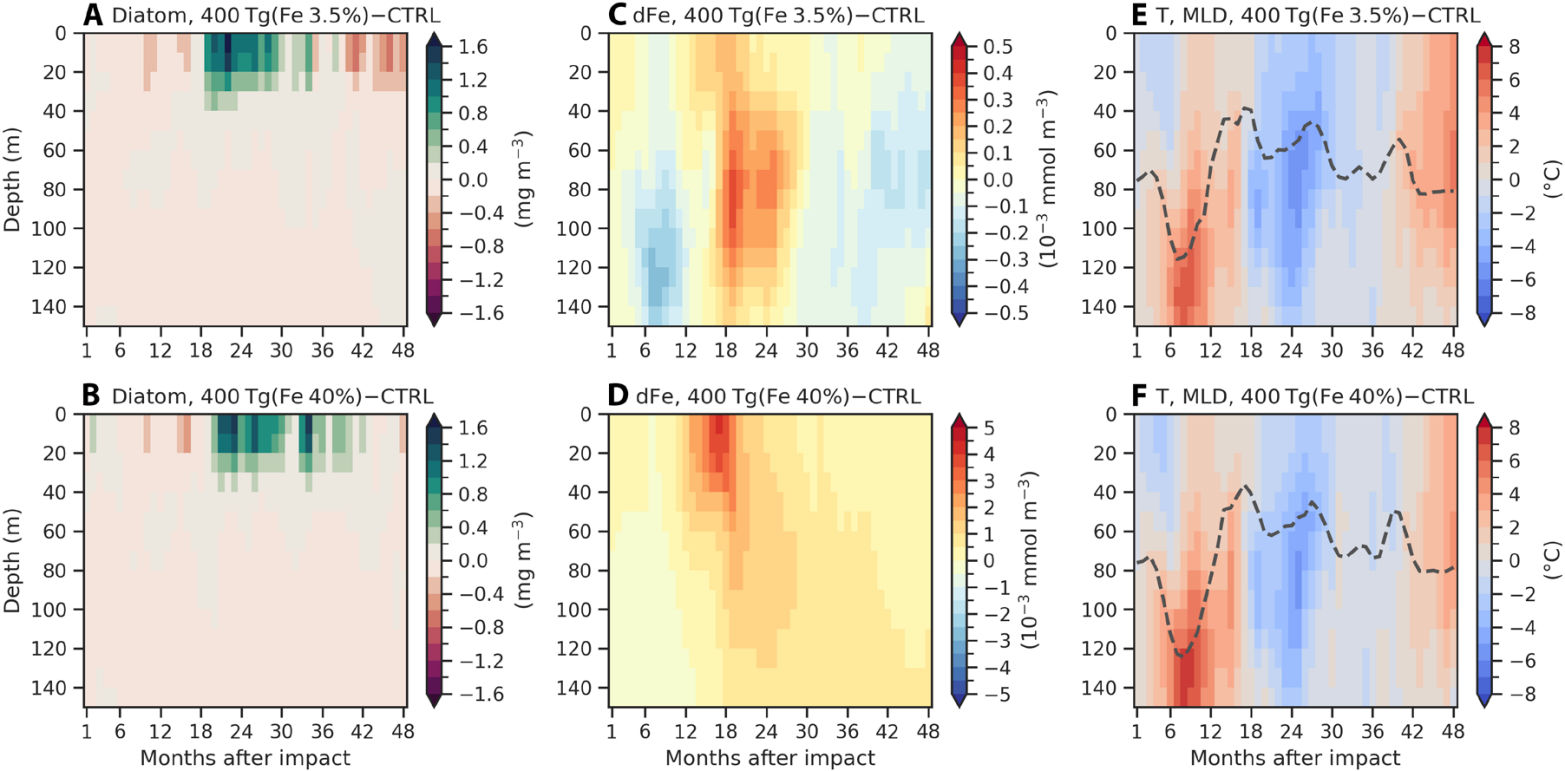
Drivers of diatom blooms over the eastern equatorial Pacific in two 400 Tg of dust simulations. Vertical distributions of (**A**) diatom chlorophyll, (**C**) dissolved inorganic iron, and (**E**) ocean temperature (shading) and mixed-layer depth (dashed line) averaged over the eastern equatorial Pacific (5°S to 5°N, 210°E to 270°E) in the upper ocean of 150 m for 400 Tg of dust simulation with 3.5% iron content. (**B**), (**D**), and (**F**), as in (A), (C), and (E), but for 400 Tg of dust simulation with 40% iron content. Note the different color scales in (C) and (D).

In addition to the Southern Ocean, we see a massive increase in marine NPP in the eastern equatorial Pacific (5°S to 5°N, 210°E to 270°E) (Fig. 4D). There are positive diatom chlorophyll concentration anomalies of up to 1.65 μg/m^3^ (~16 times larger) relative to the control simulation in the upper 40 m over the eastern equatorial Pacific (Fig. 5, A and B). Diatom blooms persist from 20 to 42 months in the 40% iron case, which is longer than months 18 to 34 in the 3.5% iron case. This bloom can be explained by not only the surface dust deposition between months 11 and 30 (Fig. 5, C and D) but also the development of a La Niña event (months 18 to 30) with corresponding upwelling, shoaling of the mixed layer, and entrainment of nutrients into the euphotic zone (Fig. 5, E and F). Consistent variations in ocean temperature and mixed layer depth indicate robust physical responses in the ocean to the two 400 Tg of dust simulations (Fig. 5, E and F). Note that dissolved iron concentrations are more than 10 times higher in the 40% iron simulation, which contributes to persistent iron supply and prolonged diatom blooms (Fig. 5, B and D).

### Influence of dust injection time on diatom blooms

**Fig. 6.**
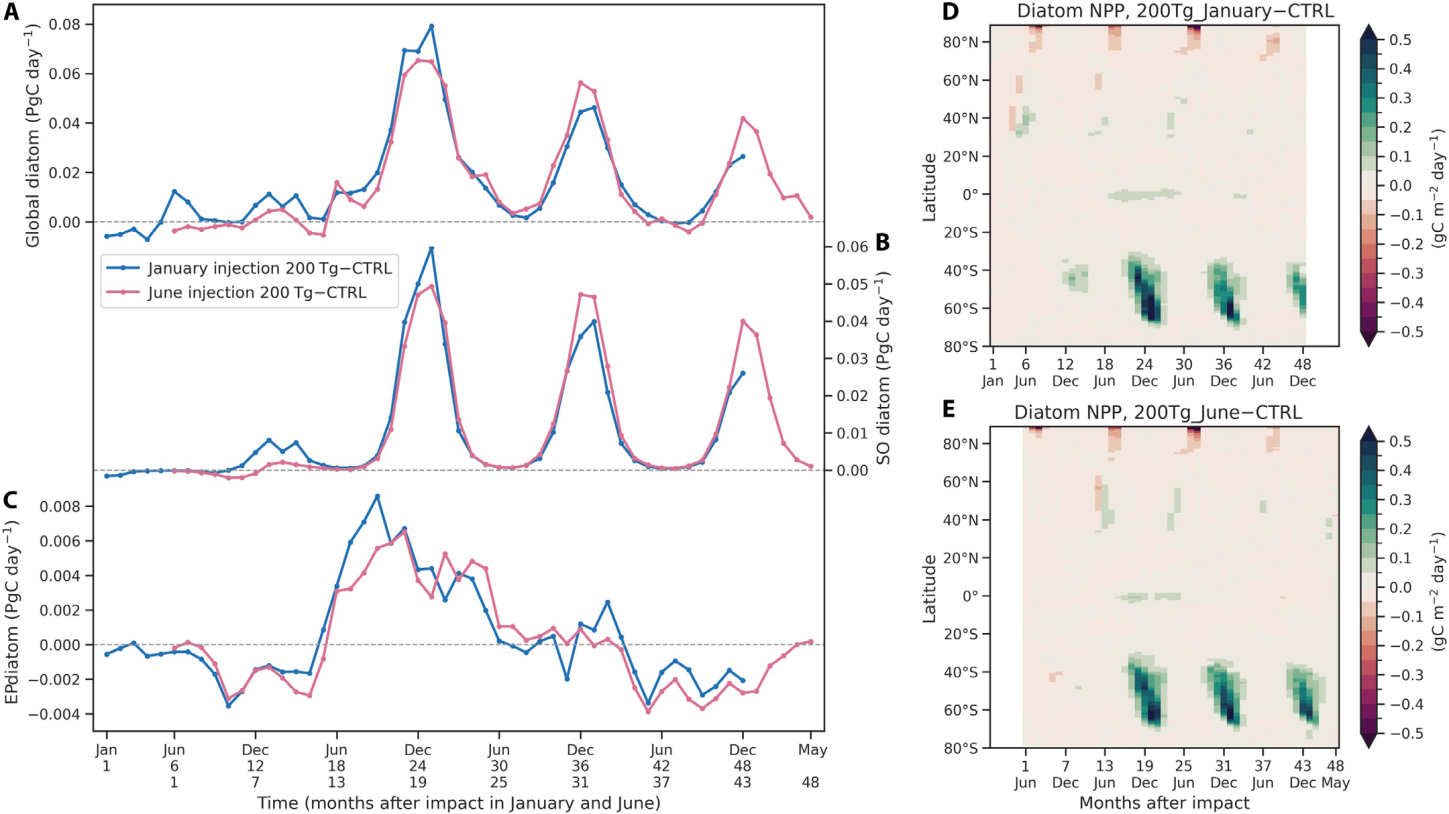
Responses of diatom bloom to different injection time in two 200 Tg of dust simulations. Time series of monthly diatom NPP anomalies integrated over (**A**) the globe, (**B**) Southern Ocean (south of 40°S across all longitudes), and (**C**) eastern equatorial Pacific (5°S to 5°N, 210°E to 270°E) for 48 months after the impact. Blue and pink lines indicate initial dust injections on 1 January and 1 June, respectively. The light black dashed lines represent zero values. Zonal distribution of latitudinally integrated diatom NPP anomalies for 48 months after the 200 Tg of dust injections in (**D**) January and (**E**) June.

We further compare two 200 Tg of dust simulations (3.5% iron content) with initial dust injections in boreal winter (1 January) and summer (1 June) to examine the effects of injection season on the timing of diatom blooms after the impact (see Materials and Methods). The globally integrated diatom NPP peaks in boreal winter of the second year after the impact, which is independent of the injection season of dust aerosols (Fig. 6A). However, in terms of recovery time of diatom productivity, the first peak occurs between months 18 and 20 after the impact in the June injection scenario, which is 5 months earlier than in the January injection scenario (between months 23 and 25). The earlier blooms following the June injection are also demonstrated in the Southern Ocean and the eastern equatorial Pacific (Fig. 6, B and C). Increased diatom NPP in the Southern Ocean accounts for a large proportion of the global diatom NPP anomalies, with peak values of 0.059 (0.049) PgC/day in the Southern Ocean and 0.079 (0.065) PgC/day over the globe following January (June) injections, respectively (Fig. 6B). However, relatively small amounts of diatoms over the eastern equatorial Pacific make a small contribution to the global total diatom NPP (Fig. 6C). The zonal distribution of diatom NPP anomalies indicates that diatom blooms are dominated by three peak periods between 40°S and 70°S in austral spring and summer from the second year after the impact (Fig. 6, D and E). Iron fertilization by dust deposition starting from the second year greatly alleviates the main nutrient limitation of diatom growth in the Southern Ocean. However, the timing of diatom growth is also modulated by light availability, which determines the growing season of diatoms from October to the following February in the southern mid-to-high latitudes.

## DISCUSSION

### Main conclusions

Using state-of-the-art Earth system model simulations with interactive atmospheric chemistry and land and ocean biogeochemistry, our study quantifies the global climatic and ecological effects of a Bennu-type asteroid collision with Earth (fig. S1). Medium-sized asteroid impacts, similar to other sun-blocking catastrophes including nuclear winter (*35*) and large volcanic eruptions (*21*), would cause abrupt climate cooling that leads to severe environmental consequences. Our simulations of up to 400 Tg of dust injections from the impacts of asteroids with diameters ranging from 465 to 739 m (fig. S2B; see Materials and Methods) indicate dramatic climate perturbations with maximum reductions in global mean surface temperature of up to 4°C, drops in precipitation rates by 15%, and severe ozone depletion by 32%. Dust particles with atmospheric lifetime of up to 2 years cause the dramatic “impact winter” characterized by global surface cooling and weakened hydrological cycle lasting for more than 4 years after the impact. Surface UV index calculations, which are not part of our model setup, would be necessary to further clarify the effects of ozone depletion on surface UV radiation.

The abrupt “impact winter” provides unfavorable climate conditions to plant growth and causes photosynthesis decline in terrestrial and marine ecosystems, with largest reductions of global land NPP by 36% and marine NPP by 25%. The initial reductions in ecosystem productivity on land and in the ocean would disrupt food availability and threaten global food security. After a relatively short-lived period of decline, marine phytoplankton starts to recover in the first boreal summer. Subsequently, diatom blooms persist for at least 3 years over the Southern Ocean and the eastern equatorial Pacific, due to iron fertilization from impact-induced dust deposition into the ocean and strong upwelling during La Niña conditions, respectively. Phytoplankton blooms in the marine biosphere could help to alleviate emerging food crises triggered by the reduction in terrestrial productivity, at least for several years after the impact. A brief peak of ocean productivity has also been found in previous simulations of the Chicxulub impact (*36*). The iron amount of the asteroid plays an important role in promoting diatom growth in iron-limited regions, which differs from nuclear winter and volcanic eruption scenarios. The abrupt cooling and ecosystem collapses caused by asteroid collisions would severely reduce the habitat suitability for humans, wildlife, and terrestrial ecosystems. Our simulated climatic and ecological responses to dust injections from medium-sized asteroid collisions provide the basis to quantify the possible effects of abrupt events on planetary life.

### Regional response to asteroid impact time

Asteroid impacts can take place at any time of the year. Dust aerosols are more concentrated in the winter hemisphere than in the summer hemisphere shortly after the impact due to the Brewer-Dobson circulation (fig. S3). The asymmetric distributions of stratospheric aerosols lead to different radiative forcings and associated regional climate responses in the NH and SH (*37*). The two simulations of 200 Tg of dust injections from the boreal winter (January) and summer (June) impacts suggest stronger cooling in the NH continents after the winter impact and more cooling in the SH after the summer impact (fig. S18, A to C). Summer and winter impacts cause similar precipitation responses in the tropics, with the ITCZ shifting more toward the warmer hemisphere (fig. S18, C to F). Terrestrial NPP also shows consistent responses to different impact seasons, but with stronger reductions over most land regions after the winter impact (fig. S18, G to I). The global mean impact responses are generally consistent to different impact seasons. The winter impact leads to stronger responses than the summer impact due to the different land-sea distributions in each hemisphere (fig. S19).

### Response of agricultural production

The land model includes prognostic crops and simulates the crop production of eight actively managed crop types (*38*). The crop yields are mainly concentrated in East Asia, western Europe, eastern North America, sub-Saharan Africa, and India (fig. S20A). Responses of crop yields to dust injections vary greatly in different crop areas (fig. S20B). The simulated negative anomalies in East Asia dominate the sharp decreases in global total crop yields of about 50% in the boreal late spring and early summer (fig. S20, B to D). Increases in global crop yields during the boreal autumn seasons can be attributed to the enhanced crop production in the western Europe (fig. S20, B to D). The distinct responses of crop yields are closely linked to regional variations in climatic responses and the dominant crop types.

### Marine biogeochemistry in the Southern Ocean

The marine biogeochemistry model computes silicate (SiO_3_) and phosphate (PO_4_) from surface dust flux forcing by default, assuming 30.8% silicon (Si) with a solubility of 7.5%, and 0.105% phosphorus (P) with a solubility of 15% in dust by weight, respectively. Increased iron concentrations (in units of μmol/m^3^) from impact dust deposition are mainly located at mid-to-high latitudes (fig. S21, A to C), where the background surface SiO_3_ and PO_4_ concentrations (in units of mmol/m^3^) are highly abundant (fig. S21, D and G). It indicates that additional inputs of surface SiO_3_ and PO_4_ fluxes from dust injections are too small compared to the climatological background values. Surface SiO_3_ and PO_4_ anomalies show strong decreases over the Southern Ocean and Southeast Pacific, resulting from more nutrient uptake by enhanced growth of diatoms and small phytoplankton, respectively (figs. S13 and S21). There are slight increases in the surface SiO_3_ and PO_4_ concentrations in North Atlantic and parts of North Pacific (fig. S21, D to I). However, the responses of phytoplankton production in these regions are not evident (fig. S13).

Phytoplankton biomass is determined by a balance between the growth rate affected by bottom-up factors and the loss rate controlled by top-down factors. The top-down control of zooplankton grazing is an important process to regulate phytoplankton biomass in the Southern Ocean (*39*–*41*). A lack of explicit representation of macro-zooplankton grazing likely leads to overestimated phytoplankton biomass during summer in the Southern Ocean (*39*). The marine biogeochemical model used in this study includes a simplified representation of only one zooplankton functional type (*34*), which may be insufficient to represent the higher trophic control on fast-growing diatoms. Thus, diatom bloom anomalies in austral spring and summer after the impact could be overestimated in our model simulations. Future implementation of more diverse plankton communities, such as the size-based traits model (*40*), might be critical to better predict the biogeochemical responses.

### Response of asteroid impacts for present-day conditions

To illustrate whether the response to asteroid impact depends on the background climate state (pre-industrial versus present-day), we compare two sets of experiments under the pre-industrial and present-day boundary conditions, respectively. The present-day simulations—including one control simulation and one 400 Tg of dust simulation with 40% iron content—are performed with fixed forcing at the year 2010. Figure S22 summarizes the main physical and biological responses from these simulations. The background mean climate state has changed dramatically since the pre-industrial era due to anthropogenic CO_2_ emissions and corresponding greenhouse warming and ocean acidification (fig. S22). Global warming and CO_2_ fertilization have substantial impacts on terrestrial vegetation production, with an increase of 9.48 PgC/year (~25%) in global annual mean NPP above pre-industrial levels (fig. S22D). However, the global marine phytoplankton productivity largely remains unchanged, suggesting small direct effects of global warming on marine phytoplankton biomass (fig. S22H). Although the background climate conditions change considerably in response to increased CO_2_ concentrations, the relative changes (i.e., anomalies) of the physical and biological responses to dust injections from asteroid impacts remain comparable under different boundary conditions (fig. S23).

### Limitations of our model simulations

It is worth noting that there are inevitable limitations about our model simulations and experimental setup. CESM2/WACCM6, similar to other modern climate models, is not designed and optimized to simulate the effects of extreme aerosol injections (*42*). Extreme perturbations by massive dust injections in the stratosphere challenge the numerical stability of the atmosphere model. Although several changes have been made to model dynamics and physics based on suggestions from previous studies (*11*, *13*, *22*), WACCM6 exhibits highly unstable behavior in the stratosphere and cannot successfully handle large dust loadings of more than 500 Tg (see more details in Supplementary Text). Moreover, the response of the global climate system depends strongly on the total amount and spatial distribution of initial dust perturbations that are insufficiently constrained (see Materials and Methods). A numerical model needs to be developed to simulate the dust nanoparticle formation in the impact plume. Climate models also need to be improved to resolve the physics and chemistry of the impact-generated dust plume in the upper atmosphere.

We only focus on the asteroid collision with the Earth’s land surface, which is less likely compared to an impact into the ocean because about 70% of Earth’s surface is covered by ocean. We assume that asteroids with diameters smaller than 1 km would not penetrate through the average depth of the ocean. An oceanic impact would—apart from massive tsunamis—inject large amounts of water vapor rather than climate-active aerosols such as dust, soot, and sulfur into the atmosphere (*16*). Previous simulations suggest that massive water vapor from the oceanic impacts of 1-km-sized asteroids could cause a multi-year global ozone depletion in the upper atmosphere (*17*).

Dust emissions from 1-km-sized asteroid impacts are suggested to cover 50% of Earth (*16*). The impacts of asteroids with diameters less than 1 km would inject dust particles over much smaller regions around the impact site. However, the regional injection area remains uncertain and can only be properly estimated from nonhydrostatic hydrocode simulations of asteroid impact plumes (*43*, *44*). To demonstrate the sensitivity to the specifics of regional dust injections, we need to perform more sensitivity scenarios in various impact sites and deal with accompanying issues of model instability arising from more confined dust distribution and aspects related to local geology, such as chemical composition and sedimentary thickness. Previous studies suggest that the climate impact of volcanic eruptions is largely influenced by eruption season and latitude instead of eruption longitude (*37*) because strong zonal winds in the stratosphere can cause quick mixing and homogenization of aerosols in the zonal direction (*45*). Motivated by these results, we injected dust particles as an initial condition over half of Earth in the Western Hemisphere across all latitudes, which would have similar results of a globally distributed emission scenario.

In addition to dust nanoparticles that have been explored in our study, further work should also consider soot emissions from wildfires ignited by impact spherules (*11*), sulfur and CO_2_ releases from target evaporites (*46*), and the shocked minerals in the ejecta curtain (*47*). The “impact winter” would be intensified and prolonged if taking account other aerosols such as soot and sulfur. Soot emissions from the 1-km-sized asteroid impact on land are estimated to be up to 28 Tg over a limited region with an area of 4 × 10^4^ km^2^ (*16*). Previous WACCM4 simulations of nuclear winter scenarios suggest that soot emissions of 5, 16.1, and 27.3 Tg in Pakistan and India cause global cooling up to 1.25° to ~4°C after the conflict, respectively (*35*). The prolonged surface cooling could generate sustained El Niño-like responses for up to 7 years after the nuclear conflict (*22*). Extreme heating from solar absorption by soot particles greatly exacerbates ozone loss in the stratosphere (*48*). Strong stratospheric heating and surface cooling would alter the circulation patterns, such as enhanced stratospheric polar vortex and positive North Atlantic Oscillation (*49*). The estimated sulfur emissions are up to 44 Tg following the 1-km-sized asteroid impacts but largely depend on the target rocks at the impact site (*16*). Previous work on the Chicxulub impact considers dust, soot, and sulfur emissions in isolation due to a lack of internal mixing of aerosols in the model configuration (*13*). It remains unclear how the climate would respond to the interactions of different aerosols. In contrast to the aerosol-driven short-term cooling, climate-active gases such as CO_2_ emissions could cause long-term warming after the impact (*46*).

## MATERIALS AND METHODS

### Impact-generated dust forcing

It is difficult to accurately quantify the aerosol forcings from medium-sized asteroid impacts on land due to a lack of observational records. Recent studies (*16*) summarized the ejecta estimations from the Chicxulub impact debris layer and extrapolated a set of initial conditions for modeling the 1-km-sized asteroid impact on land. The fine-grained ejecta consists of nanometer-sized dust particles (1 × 10^12^ kg), submicron clastics (2.6 × 10^10^ kg), soot (2.8 × 10^10^ kg), and sulfur (4.4 × 10^10^ kg). In our study, we focus mainly on dust nanoparticles recondensed from vaporized asteroid and target rocks in the impact plume. Dust nanoparticles constitute a substantial fraction of the total ejecta and have not been previously explored in great detail. Total amount and properties of the nanoparticles are poorly constrained. The upper limit of the estimated total dust amount from 1-km asteroid impacts on land is about 1000 Tg, which is about 35% of the total mass in the impact plume. Initial dust size is about 20 nm in diameter, roughly corresponding to the 15- to 25-nm iron-rich debris layer at the K/Pg boundary. The horizontal distribution of dust nanoparticles is assumed to be homogenous over half of the Earth in the Western Hemisphere (90°S to 90°N, 181°E to 360°; see more details in Discussion), while the vertical distribution follows a Gaussian distribution centered at 50 km with a width of 6.6 km (fig. S2A) (*13*, *16*). We assume that dust particles reach terminal velocity at the initial injection altitude.

### Model configuration and sensitivity experiments

We conduct all the experiments with the fully coupled configuration of the CESM2, which consists of ocean, atmosphere, land, sea-ice, land-ice, river, and wave models (*19*). We use the high-top version of the atmosphere model (WACCM6) with interactive chemistry. It has 70 vertical levels with a model top at ~140 km and a horizontal resolution of 0.9° (latitude) × 1.25° (longitude) (*20*). To better represent dust particles in a size-resolved way, we use the Community Aerosol and Radiation Model for Atmosphere (CARMA), a sectional aerosol microphysical model with 21 dust size bins (*50*). CARMA is fully coupled to chemistry, radiation, clouds, emissions, as well as wet and dry deposition in WACCM6 (*51*). Previous studies have used an earlier version at lower resolution of CESM1/WACCM4 to simulate the climatic effects of aerosol emissions from the Chicxulub impact event (*11*, *13*). The land surface model is the Community Land Model Version 5 (CLM5), which simulates many land surface processes such as biogeochemistry and ecosystem dynamics and has the same horizontal resolution as the atmosphere model (*52*). The land model configuration used here is the biogeochemistry setup with prognostic crops, which simulates the cycling of energy, water, momentum, carbon, nitrogen, and other trace gases. The ocean component is based on the Parallel Ocean Program Version 2 (POP2), which has a horizontal resolution of nominal 1° and 60 vertical levels (*53*). The Marine Biogeochemistry Library is used to simulate ocean biogeochemistry and marine ecosystem dynamics (*34*). It tracks the cycling of multiple elements of carbon, nitrogen, phosphorus, iron, silicon, and oxygen. There are three explicit phytoplankton functional groups (diatoms, diazotrophs, and small phytoplankton) and one zooplankton functional group (*34*). The land ice component uses the Community Ice Sheet Model version 2.1 (CISM2.1), which is active with ice evolution turned off (fixed ice sheets) in our simulations (*54*). The NOAA WaveWatch-III ocean surface wave prediction model (WW3) is run in the fully coupled system (*55*).

We perform a set of idealized sensitivity experiments to investigate the effects of different dust injections from medium-sized asteroid impacts (table S1). We examine, under pre-industrial climate background conditions, four dust perturbation scenarios with total amounts of 100, 200, 300, and 400 Tg of dust injections and one control scenario without dust injection. According to simple scaling laws for impact cratering (*16*, *44*), total dust amounts in the above four scenarios are injected by asteroids with diameters of 465, 586, 671, and 739 m in maximum, respectively (fig. S2B). To explore the effect of iron-rich dust deposition on marine ecosystems, we conduct an additional 400 Tg of dust simulation by replacing the iron content of dust from default 3.5 to 40%, capturing the estimated range for certain types of asteroids (*32*). Dust perturbations are injected over 1 day on 1 January as initial conditions in the above-mentioned five dust simulations. Because dust dispersion is dominated by the seasonal changes in background stratospheric circulation, we conduct another additional experiment by changing the time of dust injection from 1 January to 1 June for the 200 Tg of dust case. All the above simulations are initialized from 1850 CE pre-industrial control conditions (greenhouse gases, land use, and orbital parameters) and run for 48 months. To investigate the response to asteroid impacts for present-day climates, we have also conducted another set of experiments with one dust simulation and a control simulation under present-day boundary conditions. More details about the model experiments are described in Supplementary Text and table S1. The anomalies in this study are calculated by subtracting the corresponding values from the control run starting from the same initial conditions as the dust runs.

## References

[1] C. R. Chapman, The hazard of near-Earth asteroid impacts on earth. Earth Planet. Sci. Lett. 222, 1–15 (2004).

[2] D. Perna, M. A. Barucci, M. Fulchignoni, The near-Earth objects and their potential threat to our planet. Astron. Astrophys. Rev. 21, 1–28 (2013).

[3] NASA, Discovery Statistics (JPL Center for NEO Studies, 2024); https://cneos.jpl.nasa.gov/stats/.

[4] M. Schmieder, D. A. Kring, Earth’s impact events through geologic time: A list of recommended ages for terrestrial impact structures and deposits. Astrobiology 20, 91–141 (2020). 31880475 10.1089/ast.2019.2085 PMC6987741

[5] D. Farnocchia, S. R. Chesley, Y. Takahashi, B. Rozitis, D. Vokrouhlicky, B. P. Rush, N. Mastrodemos, B. M. Kennedy, R. S. Park, J. Bellerose, D. P. Lubey, D. Velez, A. B. Davis, J. P. Emery, J. M. Leonard, J. Geeraert, P. G. Antreasian, D. S. Lauretta, Ephemeris and hazard assessment for near-Earth asteroid (101955) Bennu based on OSIRIS-REx data. Icarus 369, 114594 (2021).

[6] O. B. Toon, K. Zahnle, D. Morrison, R. P. Turco, C. Covey, Environmental perturbations caused by the impacts of asteroids and comets. Rev. Geophys. 35, 41–78 (1997).

[7] E. Pierazzo, N. Artemieva, Local and global environmental effects of impacts on Earth. Elements 8, 55–60 (2012).

[8] T. Titus, D. Robertson, J. B. Sankey, L. Mastin, F. Rengers, A review of common natural disasters as analogs for asteroid impact effects and cascading hazards. Nat. Hazards 116, 1355–1402 (2023). 10.1007/s11069-022-05722-z PMC9900588 36776703

[9] C. Covey, S. L. Thompson, P. R. Weissman, M. C. Maccracken, Global climatic effects of atmospheric dust from an asteroid or comet impact on Earth. Glob. Planet. Change 9, 263–273 (1994).

[10] K. Kaiho, N. Oshima, K. Adachi, Y. Adachi, T. Mizukami, M. Fujibayashi, R. Saito, Global climate change driven by soot at the K-Pg boundary as the cause of the mass extinction. Sci. Rep. 6, 28427 (2016). 27414998 10.1038/srep28427 PMC4944614

[11] C. G. Bardeen, R. R. Garcia, O. B. Toon, A. J. Conley, On transient climate change at the Cretaceous−Paleogene boundary due to atmospheric soot injections. Proc. Natl. Acad. Sci. U.S.A. 114, E7415–E7424 (2017). 28827324 10.1073/pnas.1708980114 PMC5594694

[12] J. Brugger, G. Feulner, S. Petri, Baby, it’s cold outside: Climate model simulations of the effects of the asteroid impact at the end of the Cretaceous. Geophys. Res. Lett. 44, 419–427 (2017).

[13] C. R. Tabor, C. G. Bardeen, B. L. Otto-Bliesner, R. R. Garcia, O. B. Toon, Causes and climatic consequences of the impact winter at the Cretaceous-Paleogene boundary. Geophys. Res. Lett. 47, e60121 (2020).

[14] C. K. Junium, A. L. Zerkle, J. D. Witts, L. C. Ivany, T. E. Yancey, C. Liu, M. W. Claire, Massive perturbations to atmospheric sulfur in the aftermath of the Chicxulub impact. Proc. Natl. Acad. Sci. U.S.A. 119, e2119194119 (2022). 35312339 10.1073/pnas.2119194119 PMC9168947

[15] C. B. Senel, P. Kaskes, O. Temel, J. Vellekoop, S. Goderis, R. DePalma, M. A. Prins, P. Claeys, Ö. Karatekin, Chicxulub impact winter sustained by fine silicate dust. Nat. Geosci. 16, 1033–1040 (2023).

[16] O. B. Toon, C. Bardeen, R. Garcia, Designing global climate and atmospheric chemistry simulations for 1 and 10km diameter asteroid impacts using the properties of ejecta from the K-Pg impact. Atmos. Chem. Phys. 16, 13185–13212 (2016).

[17] E. Pierazzo, R. R. Garcia, D. E. Kinnison, D. R. Marsh, J. Lee-Taylor, P. J. Crutzen, Ozone perturbation from medium-size asteroid impacts in the ocean. Earth Planet. Sci. Lett. 299, 263–272 (2010).

[18] C. Bardeen, R. R. Garcia, O. B. Toon, B. L. Otto-Bliesner, E. T. Wolf, paper presented at the American Geophysical Union, Fall Meeting 2015, 14 December 2015.

[19] G. Danabasoglu, J.-F. Lamarque, J. Bacmeister, D. A. Bailey, A. K. DuVivier, J. Edwards, L. K. Emmons, J. Fasullo, R. Garcia, A. Gettelman, C. Hannay, M. M. Holland, W. G. Large, P. H. Lauritzen, D. M. Lawrence, J. T. M. Lenaerts, K. Lindsay, W. H. Lipscomb, M. J. Mills, R. Neale, K. W. Oleson, B. Otto-Bliesner, A. S. Phillips, W. Sacks, S. Tilmes, L. van Kampenhout, M. Vertenstein, A. Bertini, J. Dennis, C. Deser, C. Fischer, B. Fox-Kemper, J. E. Kay, D. Kinnison, P. J. Kushner, V. E. Larson, M. C. Long, S. Mickelson, J. K. Moore, E. Nienhouse, L. Polvani, P. J. Rasch, W. G. Strand, The Community Earth System Model version 2 (CESM2). J. Adv. Model. Earth Syst. 12, e2019MS001916 (2020).

[20] A. Gettelman, M. J. Mills, D. E. Kinnison, R. R. Garcia, A. K. Smith, D. R. Marsh, S. Tilmes, F. Vitt, C. G. Bardeen, J. McInerny, H.-L. Liu, S. C. Solomon, L. M. Polvani, L. K. Emmons, J.-F. Lamarque, J. H. Richter, A. S. Glanville, J. T. Bacmeister, A. S. Phillips, R. B. Neale, I. R. Simpson, A. K. DuVivier, A. Hodzic, W. J. Randel, The Whole Atmosphere Community Climate Model version 6 (WACCM6). J. Geophys. Res. Atmos. 124, 12380–12403 (2019).

[21] B. A. Black, J.-F. Lamarque, D. R. Marsh, A. Schmidt, C. G. Bardeen, Global climate disruption and regional climate shelters after the Toba supereruption. Proc. Natl. Acad. Sci. U.S.A. 118, e2013046118 (2021). 34230096 10.1073/pnas.2013046118 PMC8307270

[22] J. Coupe, S. Stevenson, N. S. Lovenduski, T. Rohr, C. S. Harrison, A. Robock, H. Olivarez, C. G. Bardeen, O. B. Toon, Nuclear Niño response observed in simulations of nuclear war scenarios. Commun. Earth Environ. 2, 18 (2021).

[23] C. M. Colose, A. N. LeGrande, M. Vuille, Hemispherically asymmetric volcanic forcing of tropical hydroclimate during the last millennium. Earth Syst. Dynam. 7, 681–696 (2016).

[24] S. Osipov, G. Stenchikov, K. Tsigaridis, A. N. LeGrande, S. E. Bauer, M. Fnais, J. Lelieveld, The Toba supervolcano eruption caused severe tropical stratospheric ozone depletion. Commun. Earth Environ. 2, 71 (2021).

[25] M. J. Mills, O. B. Toon, R. P. Turco, D. E. Kinnison, R. R. Garcia, Massive global ozone loss predicted following regional nuclear conflict. Proc. Natl. Acad. Sci. U.S.A. 105, 5307–5312 (2008). 18391218 10.1073/pnas.0710058105 PMC2291128

[26] P. W. Barnes, C. E. Williamson, R. M. Lucas, S. A. Robinson, S. Madronich, N. D. Paul, J. F. Bornman, A. F. Bais, B. Sulzberger, S. R. Wilson, A. L. Andrady, R. L. McKenzie, P. J. Neale, A. T. Austin, G. H. Bernhard, K. R. Solomon, R. E. Neale, P. J. Young, M. Norval, L. E. Rhodes, S. Hylander, K. C. Rose, J. Longstreth, P. J. Aucamp, C. L. Ballaré, R. M. Cory, S. D. Flint, F. R. de Gruijl, D.-P. Häder, A. M. Heikkilä, M. A. K. Jansen, K. K. Pandey, T. M. Robson, C. A. Sinclair, S.-Å. Wängberg, R. C. Worrest, S. Yazar, A. R. Young, R. G. Zepp, Ozone depletion, ultraviolet radiation, climate change and prospects for a sustainable future. Nat. Sustain. 2, 569–579 (2019).

[27] J. K. Moore, S. C. Doney, K. Lindsay, Upper ocean ecosystem dynamics and iron cycling in a global three-dimensional model. Global Biogeochem. Cycles 18, 10.1029/2004GB002220 (2004).

[28] C. M. Moore, M. M. Mills, K. R. Arrigo, I. Berman-Frank, L. Bopp, P. W. Boyd, E. D. Galbraith, R. J. Geider, C. Guieu, S. L. Jaccard, T. D. Jickells, J. La Roche, T. M. Lenton, N. M. Mahowald, E. Marañón, I. Marinov, J. K. Moore, T. Nakatsuka, A. Oschlies, M. A. Saito, T. F. Thingstad, A. Tsuda, O. Ulloa, Processes and patterns of oceanic nutrient limitation. Nat. Geosci. 6, 701–710 (2013).

[29] T. Jickells, C. M. Moore, The importance of atmospheric deposition for ocean productivity. Annu. Rev. Ecol. Evol. Syst. 46, 481–501 (2015).

[30] N. M. Mahowald, A. R. Baker, G. Bergametti, N. Brooks, R. A. Duce, T. D. Jickells, N. Kubilay, J. M. Prospero, I. Tegen, Atmospheric global dust cycle and iron inputs to the ocean. Global Biogeochem. Cycles 19, 10.1029/2004GB002402 (2005).

[31] S. Myriokefalitakis, A. Ito, M. Kanakidou, A. Nenes, M. C. Krol, N. M. Mahowald, R. A. Scanza, D. S. Hamilton, M. S. Johnson, N. Meskhidze, J. F. Kok, C. Guieu, A. R. Baker, T. D. Jickells, M. M. Sarin, S. Bikkina, R. Shelley, A. Bowie, M. M. G. Perron, R. A. Duce, Reviews and syntheses: The GESAMP atmospheric iron deposition model intercomparison study. Biogeosciences 15, 6659–6684 (2018).

[32] J. A. Sanchez, V. Reddy, W. F. Bottke, A. Battle, B. Sharkey, T. Kareta, N. Pearson, D. C. Cantillo, Physical characterization of metal-rich near-Earth asteroids 6178 (1986 DA) and 2016 ED85. Planet. Sci. J. 2, 205 (2021).

[33] K. S. Johnson, Iron supply and demand in the upper ocean: Is extraterrestrial dust a significant source of bioavailable iron? Global Biogeochem. Cycles 15, 61–63 (2001).

[34] M. C. Long, J. K. Moore, K. Lindsay, M. Levy, S. C. Doney, J. Y. Luo, K. M. Krumhardt, R. T. Letscher, M. Grover, Z. T. Sylvester, Simulations with the marine biogeochemistry library (MARBL). J. Adv. Model. Earth Syst. 13, e2021MS002647 (2021).

[35] O. B. Toon, C. G. Bardeen, A. Robock, L. Xia, H. Kristensen, M. McKinzie, R. J. Peterson, C. S. Harrison, N. S. Lovenduski, R. P. Turco, Rapidly expanding nuclear arsenals in Pakistan and India portend regional and global catastrophe. Sci. Adv. 5, eaay5478 (2019). 31616796 10.1126/sciadv.aay5478 PMC6774726

[36] J. Brugger, G. Feulner, M. Hofmann, S. Petri, A pronounced spike in ocean productivity triggered by the chicxulub impact. Geophys. Res. Lett. 48, e2020GL092260 (2021).

[37] Z. Zhuo, I. Kirchner, S. Pfahl, U. Cubasch, Climate impact of volcanic eruptions: The sensitivity to eruption season and latitude in MPI-ESM ensemble experiments. Atmos. Chem. Phys. 21, 13425–13442 (2021).

[38] D. L. Lombardozzi, Y. Lu, P. J. Lawrence, D. M. Lawrence, S. Swenson, K. W. Oleson, W. R. Wieder, E. A. Ainsworth, Simulating agriculture in the community land model version 5. J.Geophys. Res. Biogeosci. 125, e2019JG005529 (2020).

[39] C. Le Quéré, E. T. Buitenhuis, R. Moriarty, S. Alvain, O. Aumont, L. Bopp, S. Chollet, C. Enright, D. J. Franklin, R. J. Geider, S. P. Harrison, A. G. Hirst, S. Larsen, L. Legendre, T. Platt, I. C. Prentice, R. B. Rivkin, S. Sailley, S. Sathyendranath, N. Stephens, M. Vogt, S. M. Vallina, Role of zooplankton dynamics for Southern Ocean phytoplankton biomass and global biogeochemical cycles. Biogeosciences 13, 4111–4133 (2016).

[40] G. Negrete-García, J. Y. Luo, M. C. Long, K. Lindsay, M. Levy, A. D. Barton, Plankton energy flows using a global size-structured and trait-based model. Prog. Oceanogr. 209, 102898 (2022).

[41] T. Rohr, A. J. Richardson, A. Lenton, M. A. Chamberlain, E. H. Shadwick, Zooplankton grazing is the largest source of uncertainty for marine carbon cycling in CMIP6 models. Commun. Earth Environ. 4, 212 (2023).

[42] S. Eastham, S. Doherty, D. Keith, J. H. Richter, L. Xia, Improving models for solar climate intervention research. Eos 102, 10.1029/2021EO156087 (2021).

[43] N. Artemieva, J. Morgan, Modeling the formation of the K–Pg boundary layer. Icarus 201, 768–780 (2009).

[44] B. C. Johnson, H. J. Melosh, Formation of spherules in impact produced vapor plumes. Icarus 217, 416–430 (2012).

[45] S. Kremser, L. W. Thomason, M. von Hobe, M. Hermann, T. Deshler, C. Timmreck, M. Toohey, A. Stenke, J. P. Schwarz, R. Weigel, S. Fueglistaler, F. J. Prata, J.-P. Vernier, H. Schlager, J. E. Barnes, J.-C. Antuña-Marrero, D. Fairlie, M. Palm, E. Mahieu, J. Notholt, M. Rex, C. Bingen, F. Vanhellemont, A. Bourassa, J. M. C. Plane, D. Klocke, S. A. Carn, L. Clarisse, T. Trickl, R. Neely, A. D. James, L. Rieger, J. C. Wilson, B. Meland, Stratospheric aerosol—Observations, processes, and impact on climate. Rev. Geophys. 54, 278–335 (2016).

[46] N. Artemieva, J. Morgan, Expedition 364 Science Party, Quantifying the release of climate-active gases by large meteorite impacts with a case study of chicxulub. Geophys. Res. Lett. 44, 10,180–10,188 (2017).

[47] N. Artemieva, J. Morgan, Global K-Pg layer deposited from a dust cloud. Geophys. Res. Lett. 47, e2019GL086562 (2020).

[48] C. G. Bardeen, D. E. Kinnison, O. B. Toon, M. J. Mills, F. Vitt, L. Xia, J. Jägermeyr, N. S. Lovenduski, K. J. N. Scherrer, M. Clyne, A. Robock, Extreme ozone loss following nuclear war results in enhanced surface ultraviolet radiation. J. Geophys. Res. Atmos. 126, e2021JD035079 (2021).

[49] J. Coupe, A. Robock, The influence of stratospheric soot and sulfate aerosols on the Northern hemisphere wintertime atmospheric circulation. J. Geophys. Res. Atmos. 126, e2020JD034513 (2021).

[50] C. G. Bardeen, O. B. Toon, E. J. Jensen, D. R. Marsh, V. L. Harvey, Numerical simulations of the three-dimensional distribution of meteoric dust in the mesosphere and upper stratosphere. J. Geophys. Res. Atmos. 113, 10.1029/2007JD009515 (2008).

[51] S. Tilmes, M. J. Mills, Y. Zhu, C. G. Bardeen, F. Vitt, P. Yu, D. Fillmore, X. Liu, B. Toon, T. Deshler, Description and performance of a sectional aerosol microphysical model in the Community Earth System Model (CESM2). Geosci. Model Dev. 16, 6087–6125 (2023).

[52] D. M. Lawrence, R. A. Fisher, C. D. Koven, K. W. Oleson, S. C. Swenson, G. Bonan, N. Collier, B. Ghimire, L. van Kampenhout, D. Kennedy, E. Kluzek, P. J. Lawrence, F. Li, H. Li, D. Lombardozzi, W. J. Riley, W. J. Sacks, M. Shi, M. Vertenstein, W. R. Wieder, C. Xu, A. A. Ali, A. M. Badger, G. Bisht, M. van den Broeke, M. A. Brunke, S. P. Burns, J. Buzan, M. Clark, A. Craig, K. Dahlin, B. Drewniak, J. B. Fisher, M. Flanner, A. M. Fox, P. Gentine, F. Hoffman, G. Keppel-Aleks, R. Knox, S. Kumar, J. Lenaerts, L. R. Leung, W. H. Lipscomb, Y. Lu, A. Pandey, J. D. Pelletier, J. Perket, J. T. Randerson, D. M. Ricciuto, B. M. Sanderson, A. Slater, Z. M. Subin, J. Tang, R. Q. Thomas, M. Val Martin, X. Zeng, The Community Land Model version 5: Description of new features, benchmarking, and impact of forcing uncertainty. J. Adv. Model. Earth Syst. 11, 4245–4287 (2019).

[53] G. Danabasoglu, S. C. Bates, B. P. Briegleb, S. R. Jayne, M. Jochum, W. G. Large, S. Peacock, S. G. Yeager, The CCSM4 ocean component. J. Clim. 25, 1361–1389 (2012).

[54] W. H. Lipscomb, S. F. Price, M. J. Hoffman, G. R. Leguy, A. R. Bennett, S. L. Bradley, K. J. Evans, J. G. Fyke, J. H. Kennedy, M. Perego, D. M. Ranken, W. J. Sacks, A. G. Salinger, L. J. Vargo, P. H. Worley, Description and evaluation of the Community Ice Sheet Model (CISM) v2.1. Geosci. Model Dev. 12, 387–424 (2019).

[55] Q. Li, A. Webb, B. Fox-Kemper, A. Craig, G. Danabasoglu, W. G. Large, M. Vertenstein, Langmuir mixing effects on global climate: WAVEWATCH III in CESM. Ocean Model. 103, 145–160 (2016).

