# Supplementary material for: Climatic and ecological responses to Bennu-type asteroid collisions
Source: Sci Adv. 2025 Feb 5;11(6):eadq5399. doi: 10.1126/sciadv.adq5399 (PMC11797562; doi:10.1126/sciadv.adq5399)
Supplement: Supplementary file 1 — Supplementary Text Figs. S1 to S23 Table S1 [file sciadv.adq5399_sm.pdf]

Science Advances
AAAS

Supplementary Materials for

Climatic and ecological responses to Bennu-type asteroid collisions

Lan Dai and Axel Timmermann

Corresponding author: Lan Dai, landai@pusan.ac.kr; Axel Timmermann, axel@ibsclimate.org

Sci. Adv. **11**, eadq5399 (2025)
DOI: 10.1126/sciadv.adq5399

This PDF file includes:

Supplementary Text
Figs. S1 to S23
Table S1

Supplementary Text

Model configuration

We use the all-active and fully-coupled CESM2 configuration that has been contributed to the Coupled Model Intercomparison Project Phase 6 (CMIP6). Our pre-industrial (PI) simulations are restarted from previous 1850 PI control simulations supported by the National Center for Atmospheric Research (NCAR). Six PI dust simulations and one PI control simulation are conducted for forty-eight months. We also perform a 10-year spin-up simulation under present-day (PD) conditions with fixed forcings at year 2010, which is restarted from NCAR's 1850-2014 historical simulations. One PD dust simulation and a PD control simulation are then branched from the spin-up simulation. We use one control simulation and do not consider the internal variability of the background climate, which is quite small compared to the climatic responses to massive dust injections. The datasets with initial conditions can be found online (https://svn-ccsm-inputdata.cgd.ucar.edu/trunk/inputdata/cesm2_init/). All the model simulations conducted in this study are listed in Table S1.

Initial injections of massive dust exert extreme temperature perturbations in the upper atmosphere, resulting in strong numerical instability of WACCM6. We apply similar modifications to increase the model stability as performed in previous studies (*11, 13, 22*). The default dynamics timesteps, vertical remapping timesteps, and tracer advection timesteps per physics timestep are 16, 4, and 4 in WACCM6 at $0.9^{\circ} \times 1.25^{\circ}$ horizontal resolution. When the model instability occurs in the first few months, we increase the number of dynamics timesteps to 64, 128, 256, or 512 depending on how unstable the model behaves under different dust loadings. The number of vertical remapping and tracer advection timesteps is increased accordingly as the divisors of dynamics timesteps. We extend the dry convective adjustment to cover more model levels than the default 3 layers from the top of the model. We also increase the maximum number of iterations to achieve convergence in dry adiabatic adjustment from default 15 to 200. Previous studies have verified that these modifications have minimal impacts on the control simulation (*11, 13*). The modifications of dynamical substeps for model stability are applied only for up to three months following the initial dust injections. We keep the default dynamical substeps without stability modifications in the control simulation. Comparison with an additional control run with increased substeps indicates that the dynamical substepping has little discernible impact on our results.

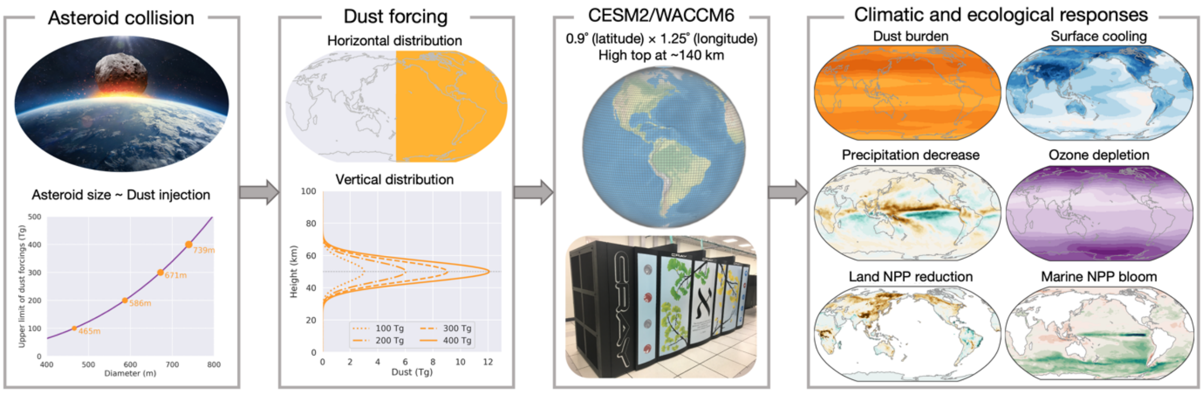

Fig. S1. Schematic figure of climatic and ecological responses to dust forcings from Bennu-type asteroid collisions with Earth.

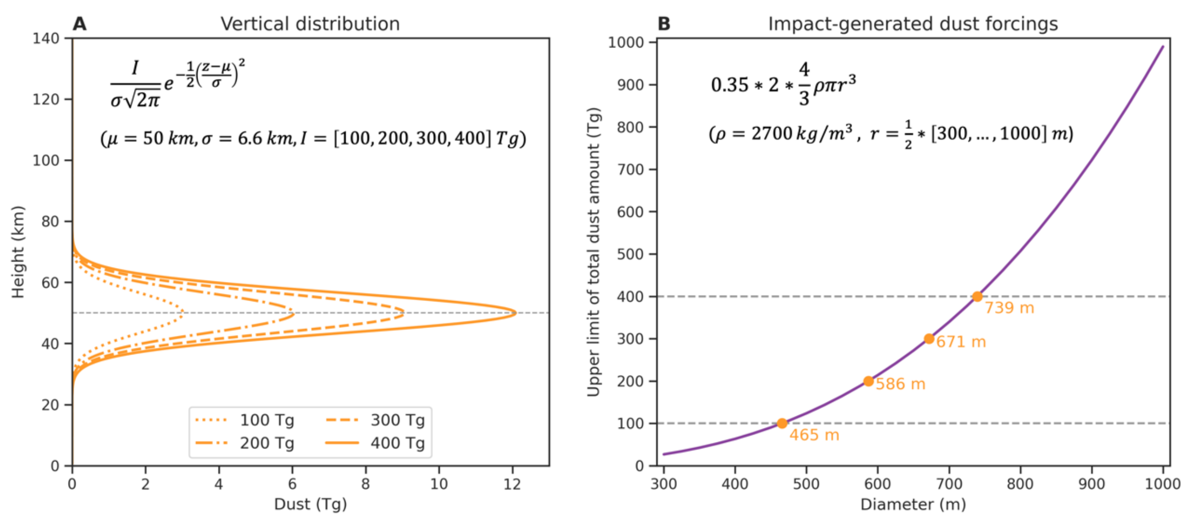

Fig. S2. (**A**) Schematic plot of vertical Gaussian distributions of the initial dust forcings centered at 50 km with a width of 6.6 km. (**B**) Upper limits of total dust amounts injected from the impacts of different-sized asteroids on land, which are assumed as 35% of the rock vapor in the impact plume (the mass of the asteroid plus an equivalent amount of target materials).

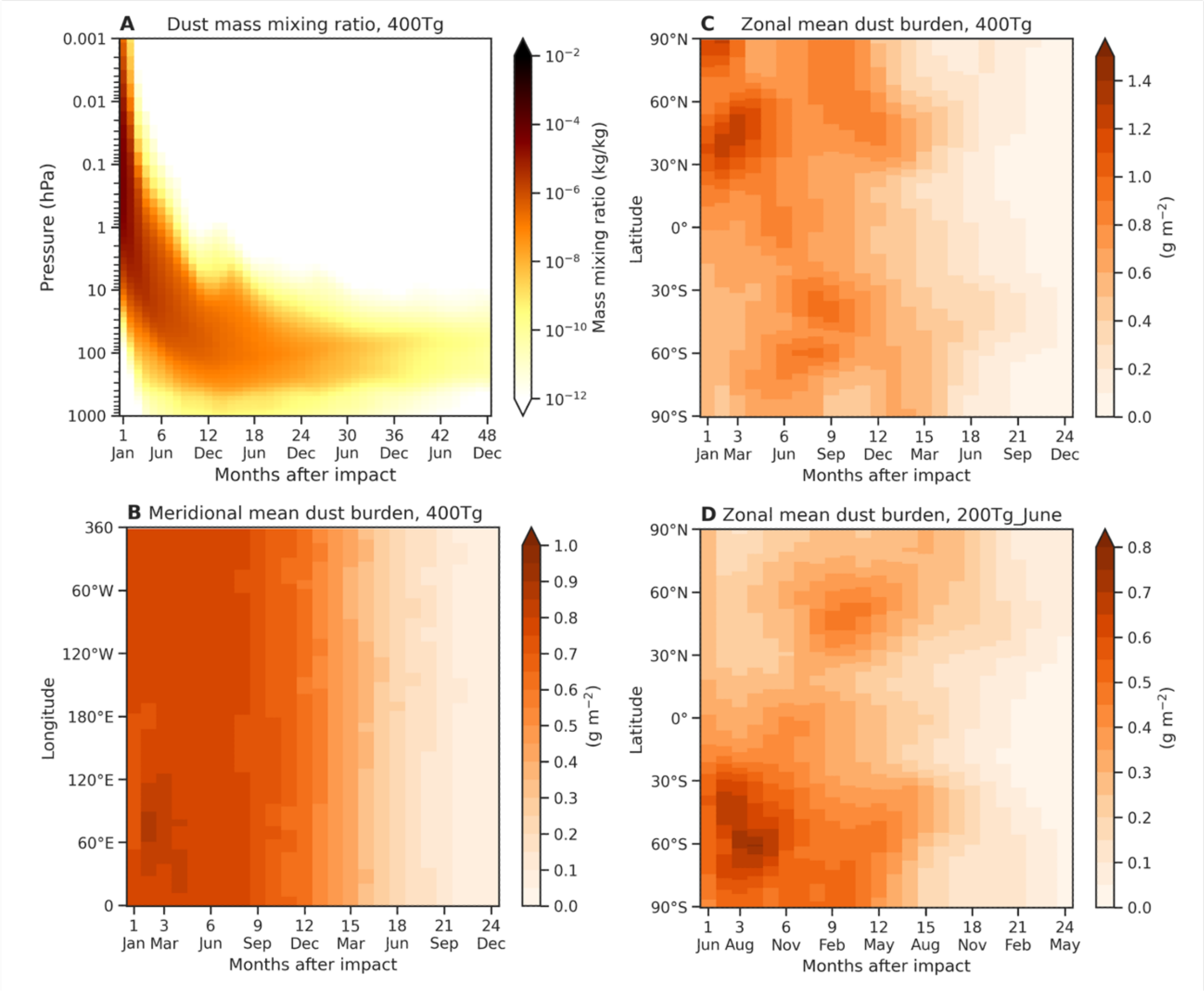

Fig. S3. Spatial evolution of dust aerosols in the atmosphere after the impact. (**A**) Vertical distribution of global mean dust mass mixing ratio over forty-eight months after the impact. (**B**) Meridional mean, and (**C**) zonal mean total column dust burden over twenty-four months in the 400-Tg simulation with dust injections in January. (**D**) Zonal mean total column dust burden over twenty-four months in the 200-Tg simulation with dust injections in June. Note the different color scales in **C**, **D**.

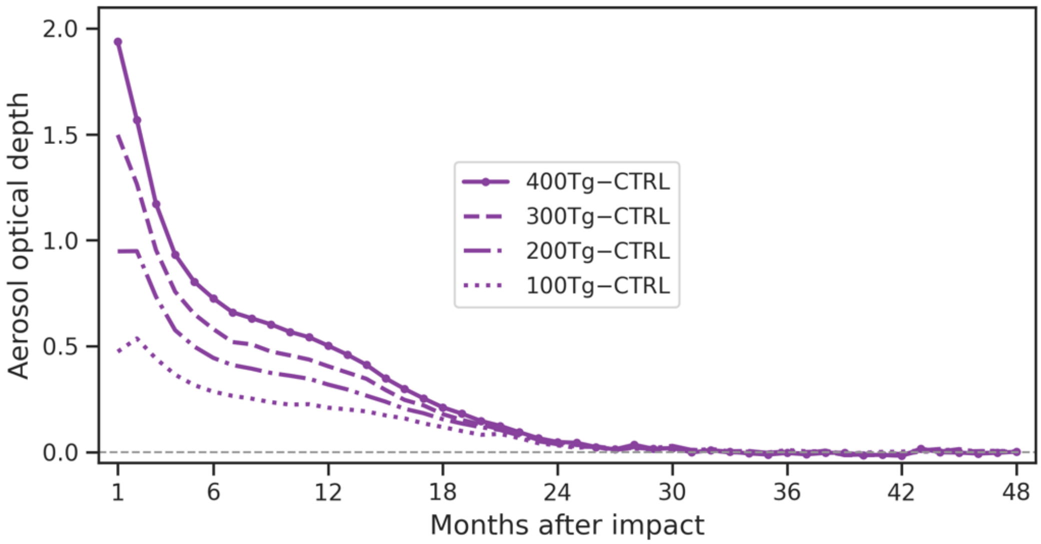

Fig. S4. Time series of globally and monthly averaged absolute changes in total aerosol optical depth in visible band relative to the control run for forty-eight months after the impact in four dust scenarios.

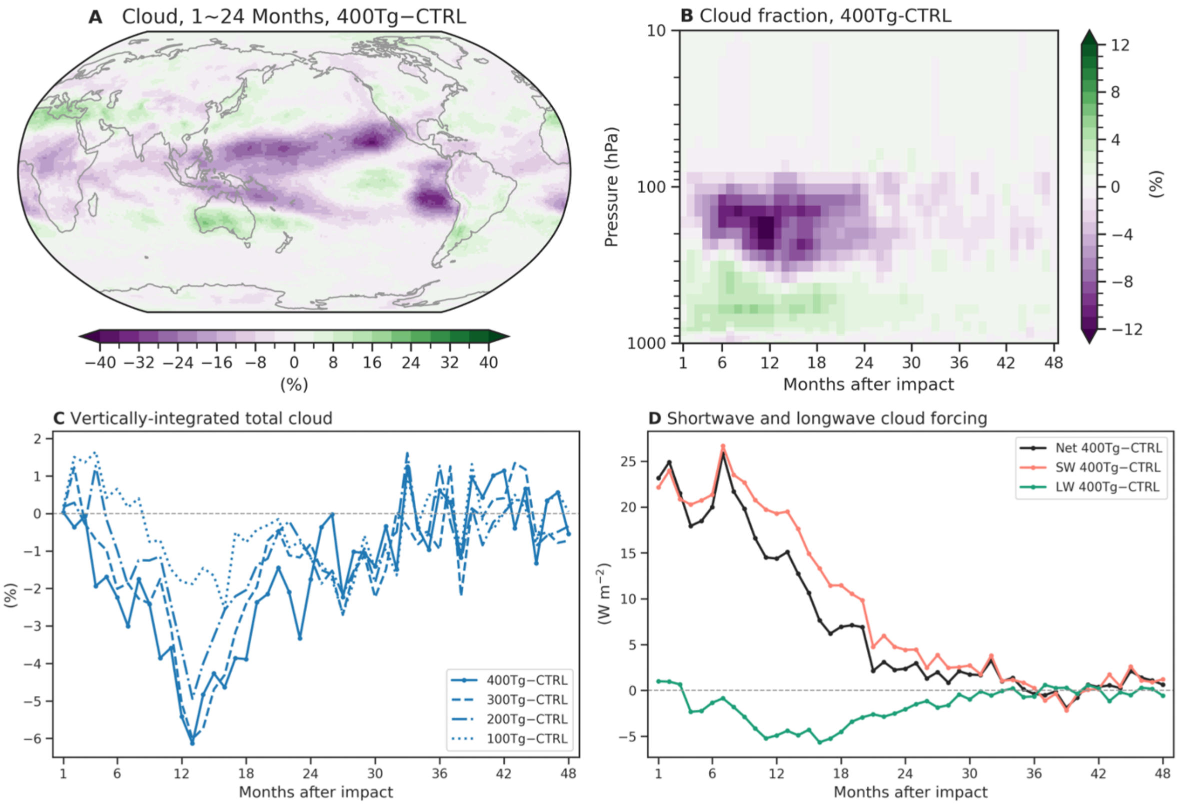

Fig. S5. (**A**) Spatial patterns of absolute changes in total cloud cover averaged from 1 to 24 months in the 400-Tg dust simulation. (**B**) Vertical distribution of global mean cloud cover in the 400-Tg dust simulation. (**C**) Time series of global mean vertically integrated total cloud in four dust simulations. (**D**) Time series of global mean shortwave and longwave cloud forcing in the 400-Tg dust simulation.

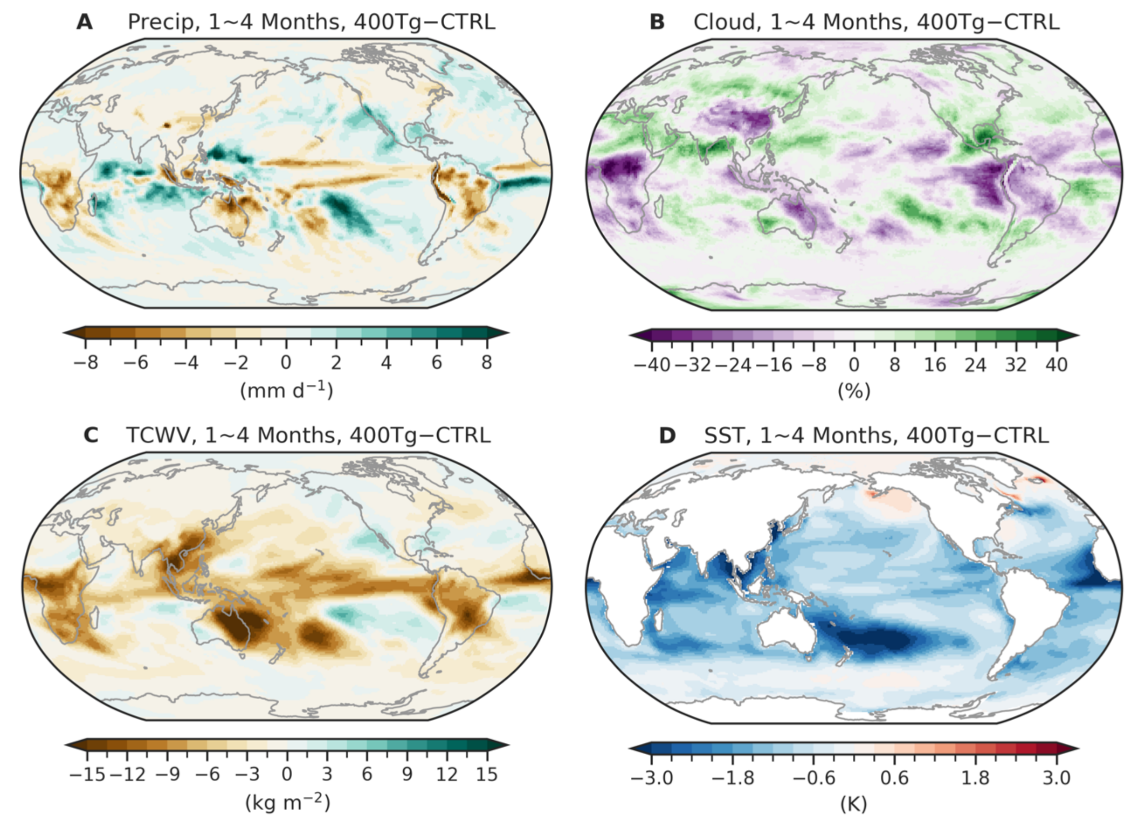

Fig. S6. Spatial patterns of changes in (**A**) precipitation, (**B**) vertically integrated cloud cover, (**C**) total column water vapor, and (**D**) sea surface temperature averaged from 1 to 4 months between the 400-Tg dust and control simulations.

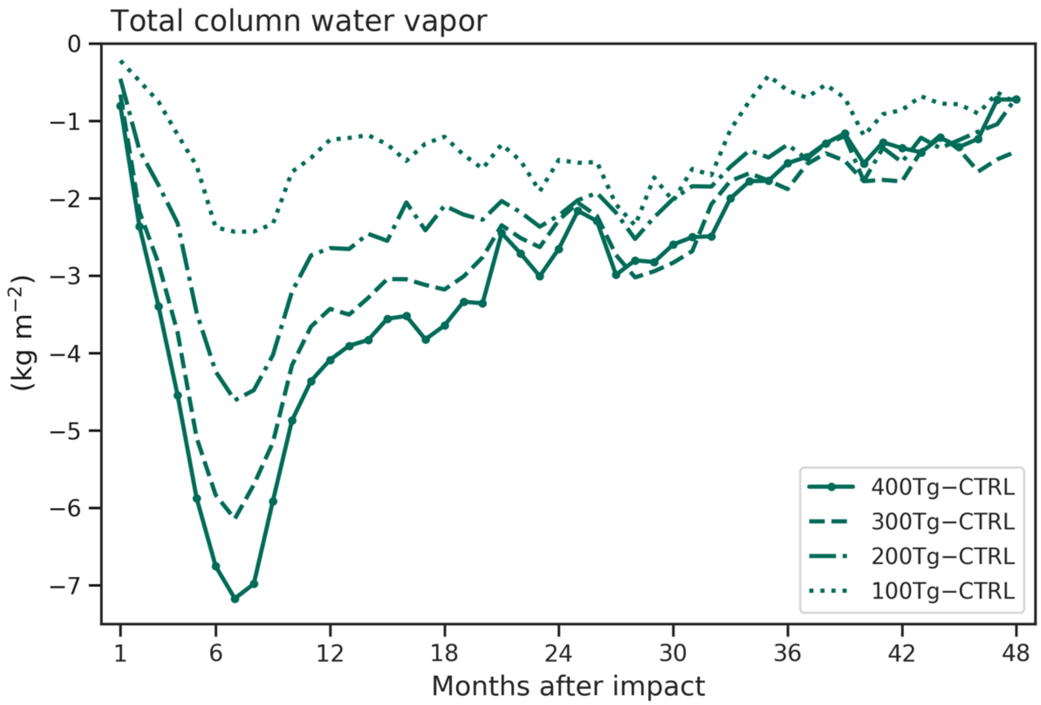

Fig. S7. Time series of the global mean vertically integrated water vapor anomalies relative to the control run for forty-eight months after the impact in four dust scenarios.

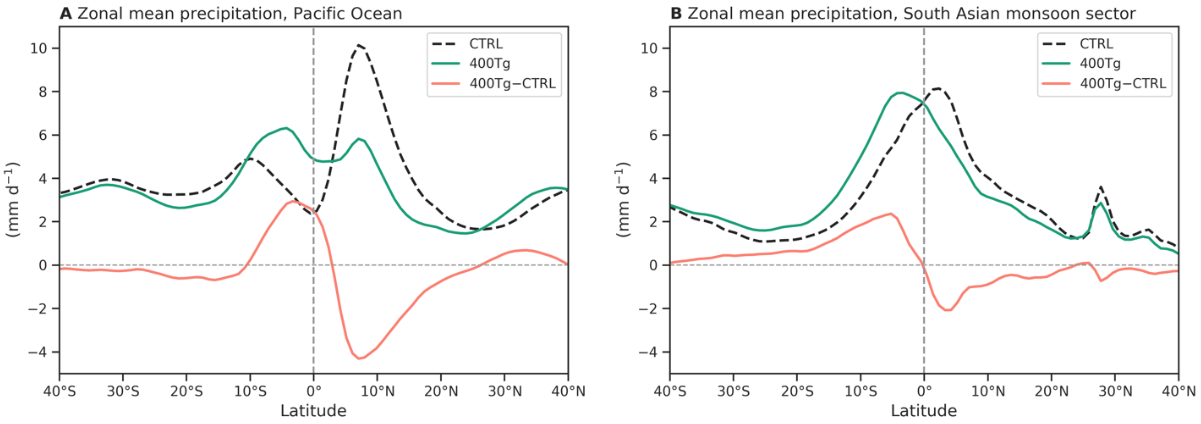

Fig. S8. Zonal mean precipitation rate over (**A**) the Pacific (160˚ E–100˚ W) and (**B**) the South Asian monsoon sector (65˚ E–95˚ E) averaged from 1 to 24 months after the impact in the 400-Tg dust and control simulations. Green, black, and pink lines represent the dust, control and their differences, respectively.

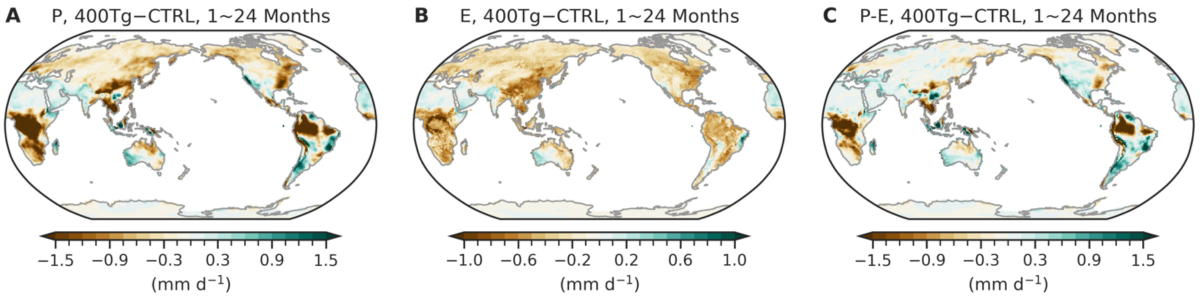

Fig. S9. Spatial patterns of changes in (**A**) precipitation, (**B**) evaporation, and (**C**) precipitation minus evaporation over land averaged from 1 to 24 months after the impact between the 400-Tg dust and the control simulations.

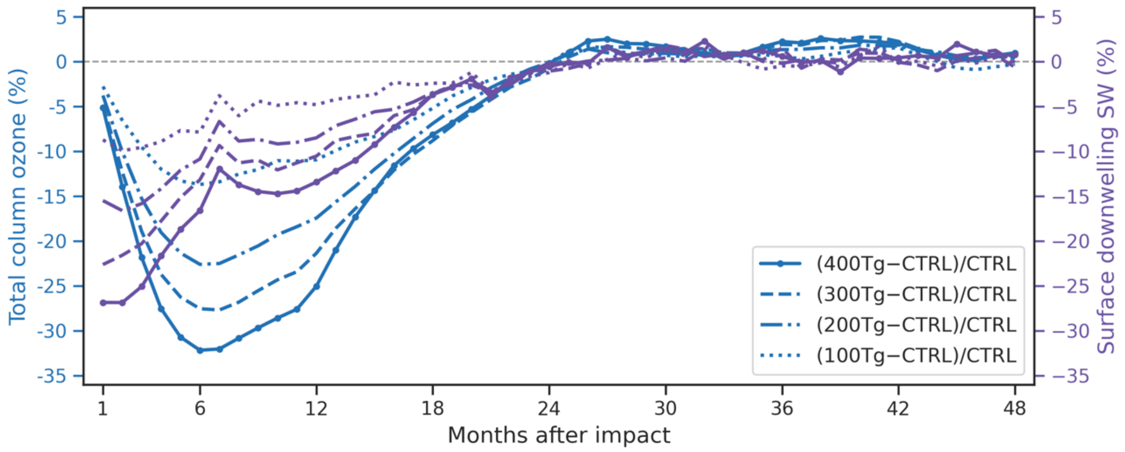

Fig. S10. Time series of globally and monthly averaged percentage changes in total column zone (blue lines) and surface downwelling solar flux (purple lines) relative to the control simulation over forty-eight months in four dust scenarios.

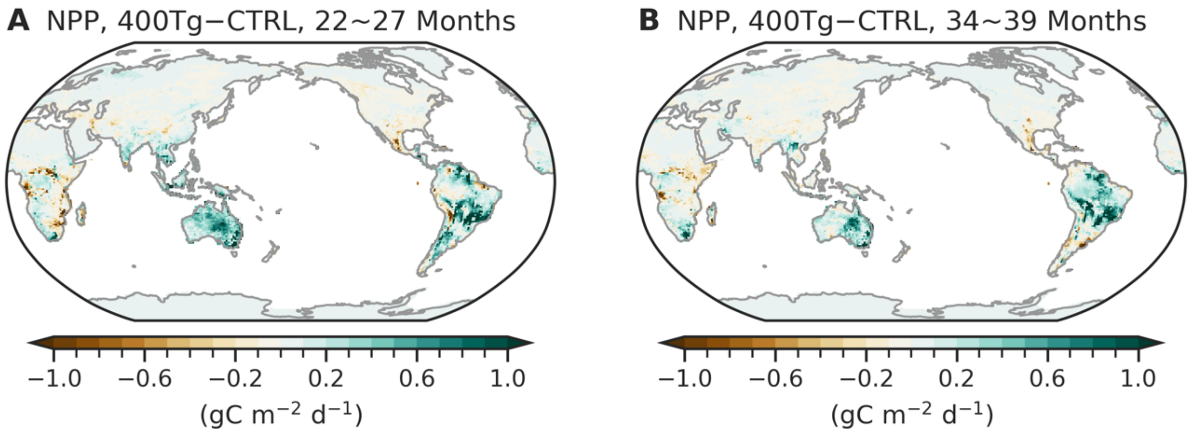

Fig. S11. Spatial patterns of land NPP anomalies averaged in (**A**) months 22-27 and (**B**) months 34-39 after the impact between the 400-Tg dust and the control simulations.

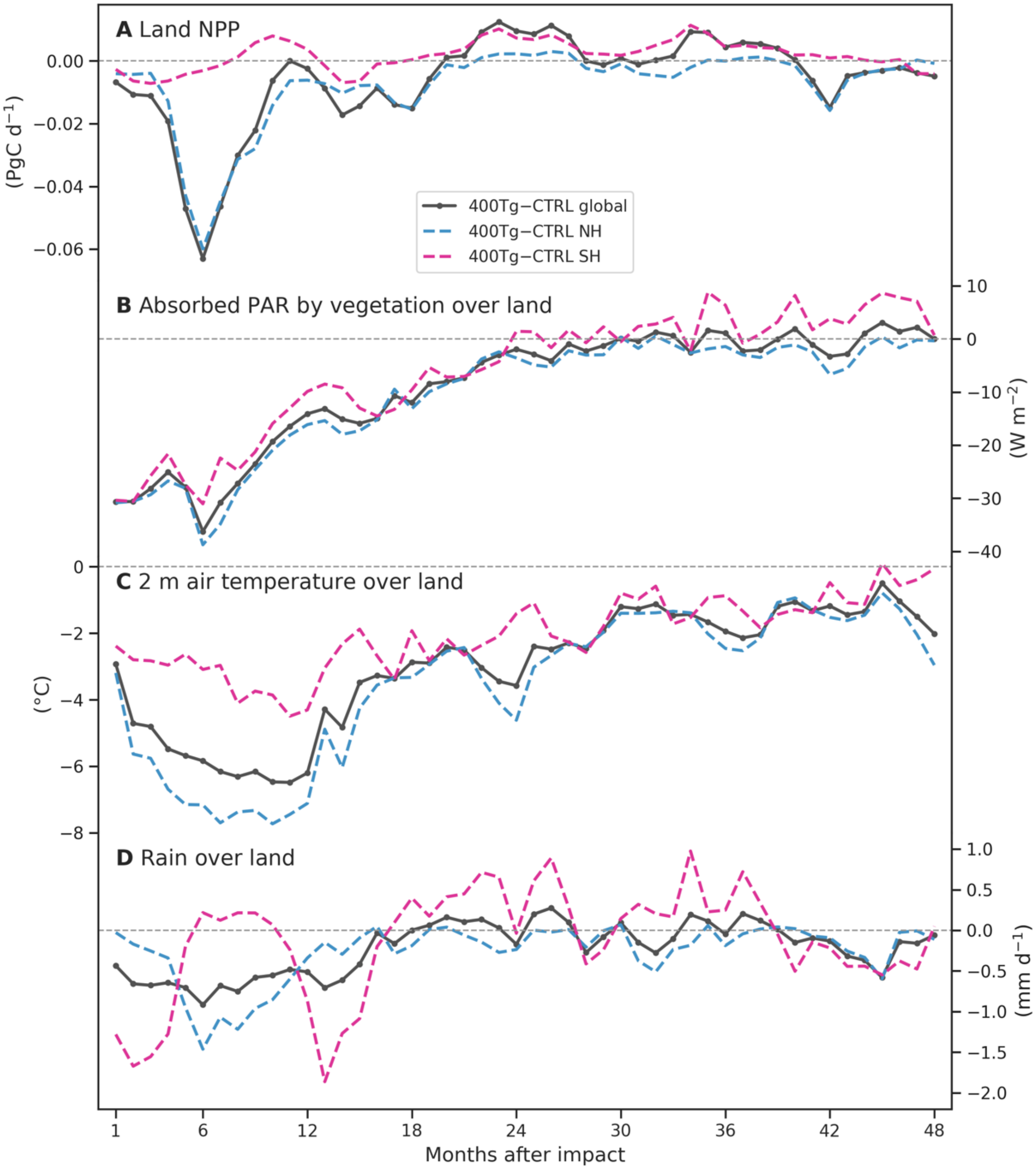

Fig. S12. Time series of anomalies in (**A**) total land NPP, and (**B**) absorbed photosynthetically active radiation (PAR) by vegetation, (**C**) 2 m air temperature, and (**D**) rain averaged over land for forty-eight months after the impact. Black, blue, and magenta lines represent globe, Northern Hemisphere (NH, 0˚-90˚N), and Southern Hemisphere (SH, 90˚S-0˚), respectively.

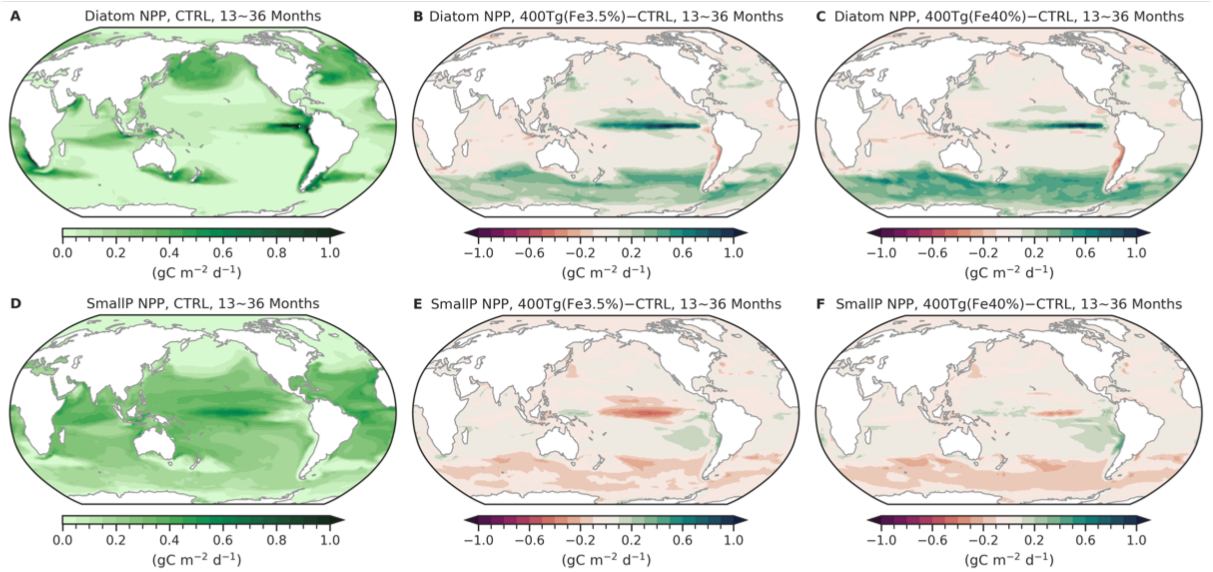

Fig. S13. Spatial patterns of (**A, B, C**) diatom NPP, and (**D, E, F**) small phytoplankton NPP averaged from 13 to 36 months after the impact for (**A, D**) the control simulation, (**B, E**) and (**C, F**) the anomalies of two 400-Tg dust simulations with 3.5% and 40% iron contents relative to the control simulation, respectively.

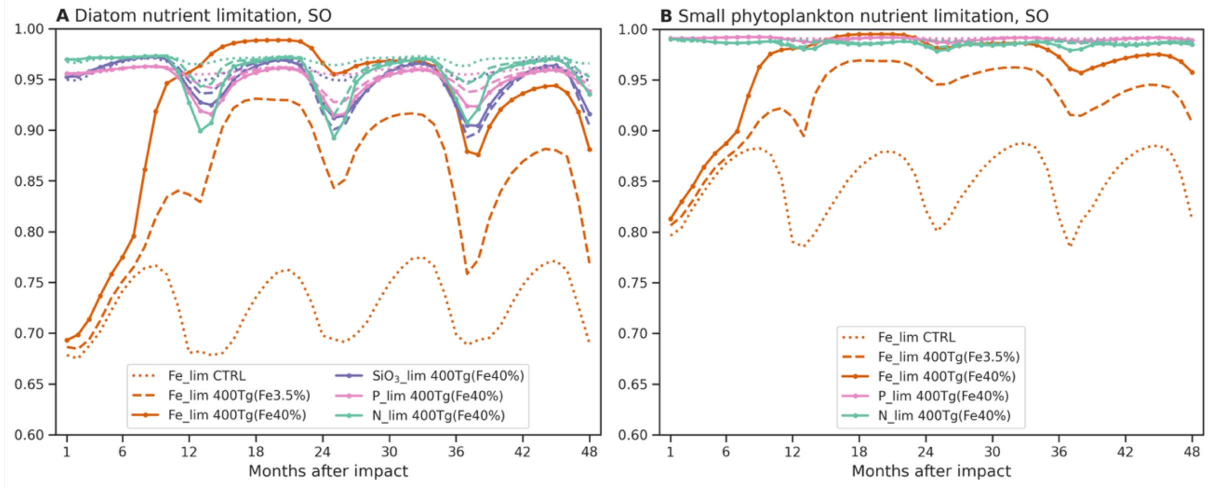

Fig. S14. Nutrient limitation (unitless values ranging from 0-1) on growth rates of (**A**) diatoms, and (**B**) small phytoplankton averaged over the Southern Ocean (South of 40°S across all longitudes). The control and two 400-Tg dust simulations with 3.5% and 40% iron contents are represented with dotted, dashed, and solid lines, respectively. Nutrient limitation is computed as carbon biomass weighted means of the top 100 m according to Michaelis-Menten kinetics (see equations 8-11 in (*34*) for more details). Lower values mean more limiting for phytoplankton growth. The most limiting nutrient has the minimum value.

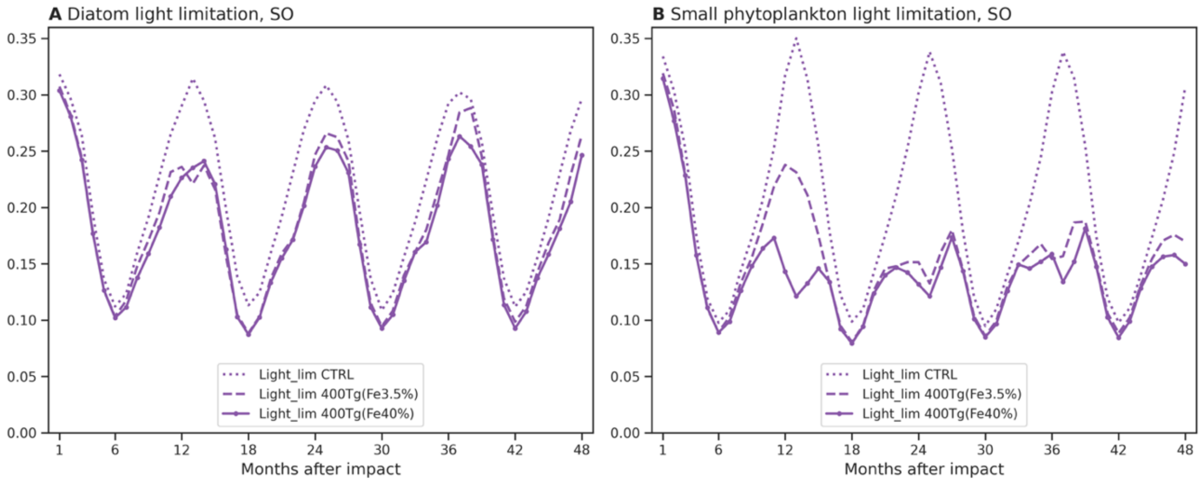

Fig. S15. Light limitation on growth rates of (**A**) diatoms, and (**B**) small phytoplankton averaged over the Southern Ocean (South of 40˚S across all longitudes) in the top 100 m. Light limitation is calculated as a function of the ratio between the supply of energy for photosynthesis and the demand for growth (see equation 5 in (*34*) for more details). Non-dimensional values in this figure reflect the sensitivities to light. Lower values indicate that phytoplankton growth is more limited by light.

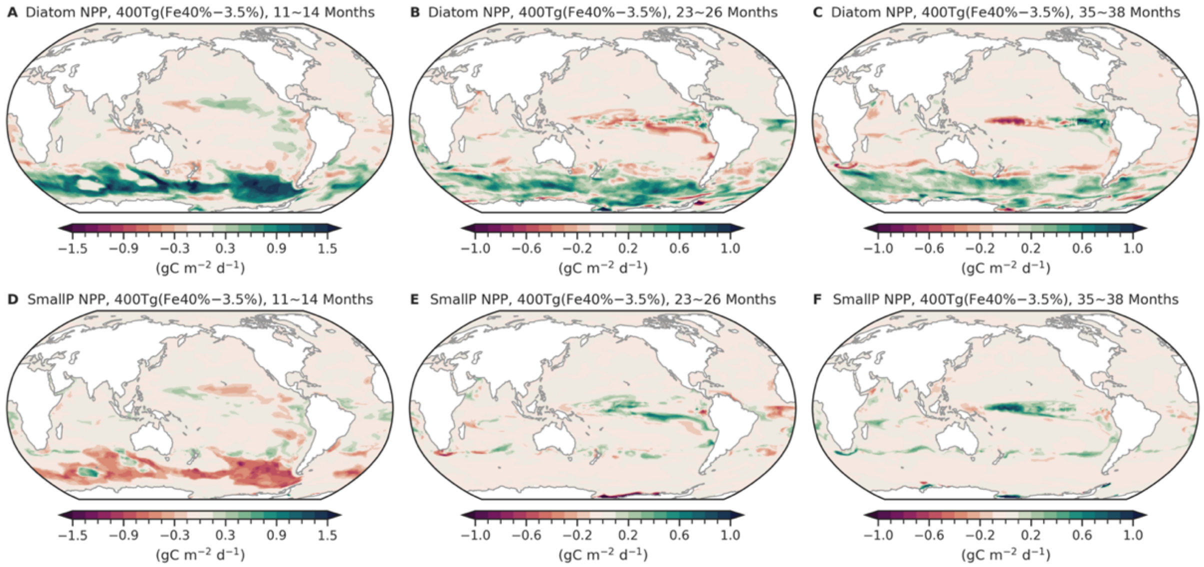

Fig. S16. Spatial patterns of (**A, B, C**) diatom NPP, and (**D, E, F**) small phytoplankton NPP averaged from November to February (months 11-14, 23-26, and 35-38) in three years after the impact for the differences between the two 400-Tg dust simulations with 40% and 3.5% iron contents.

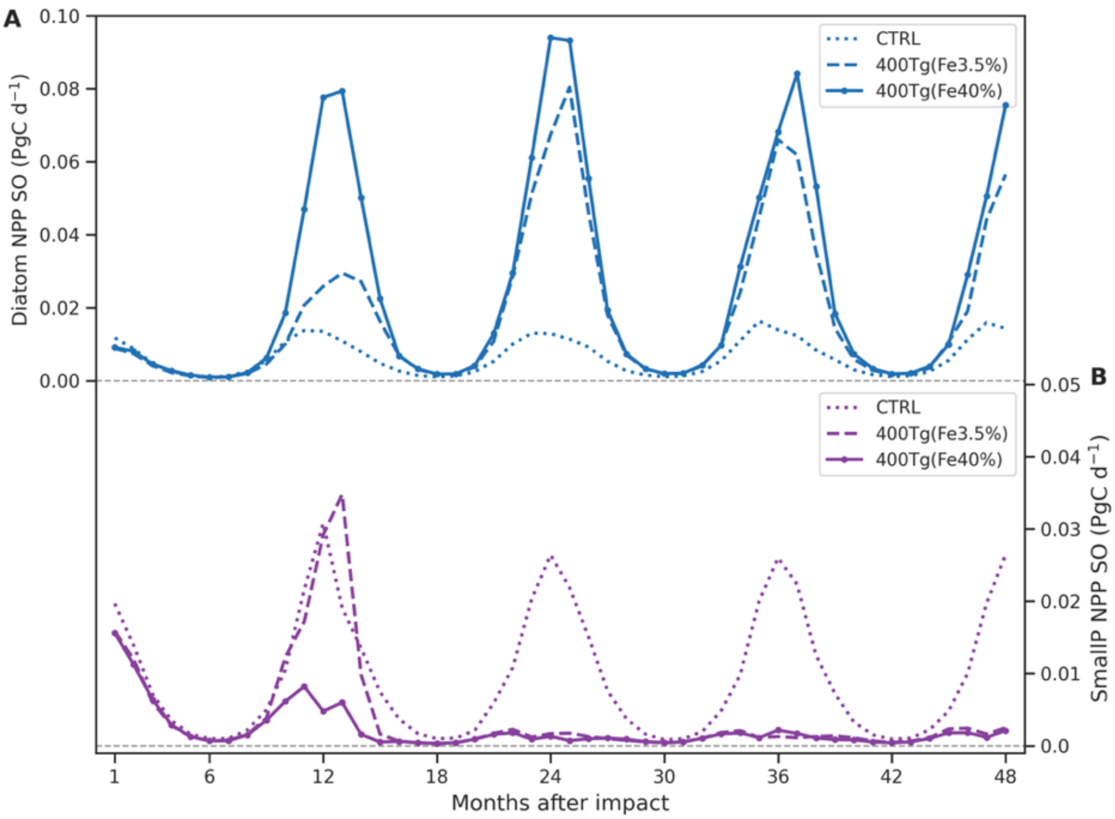

Fig. S17. Time series of monthly (**A**) diatom NPP, and (**B**) small phytoplankton NPP integrated over the Southern Ocean (South of 40°S across all longitudes) for forty-eight months after the impact. The control and two 400-Tg dust simulations with 3.5% and 40% iron are represented with dotted, dashed, and solid lines, respectively.

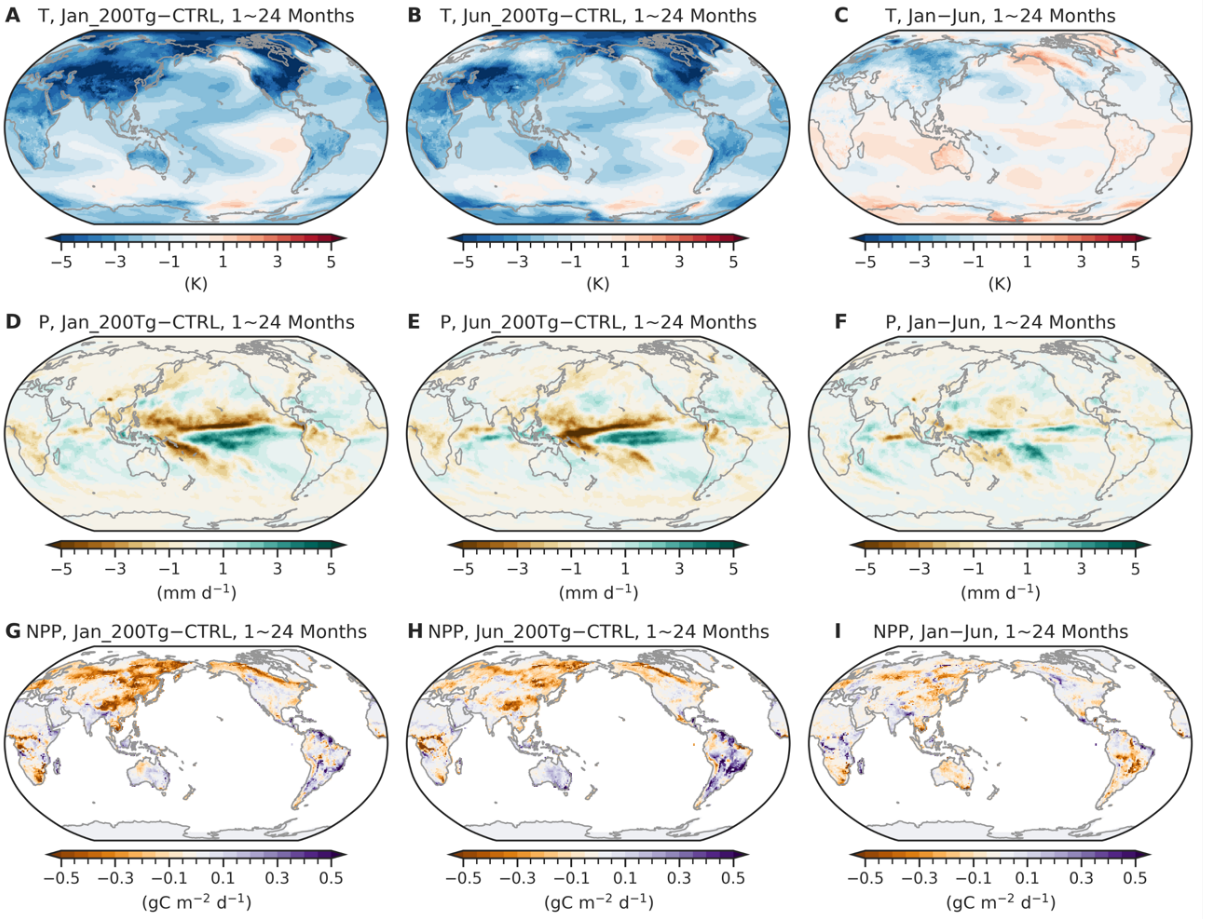

Fig. S18. Spatial patterns of (**A, B, C**) surface temperature, (**D, E, F**) precipitation, and (**G, H, I**) land NPP anomalies averaged from 1 to 24 months for (**A, D, G**) January injection, (**B, E, H**) June injection, and (**C, F, I**) the differences between January and June injections.

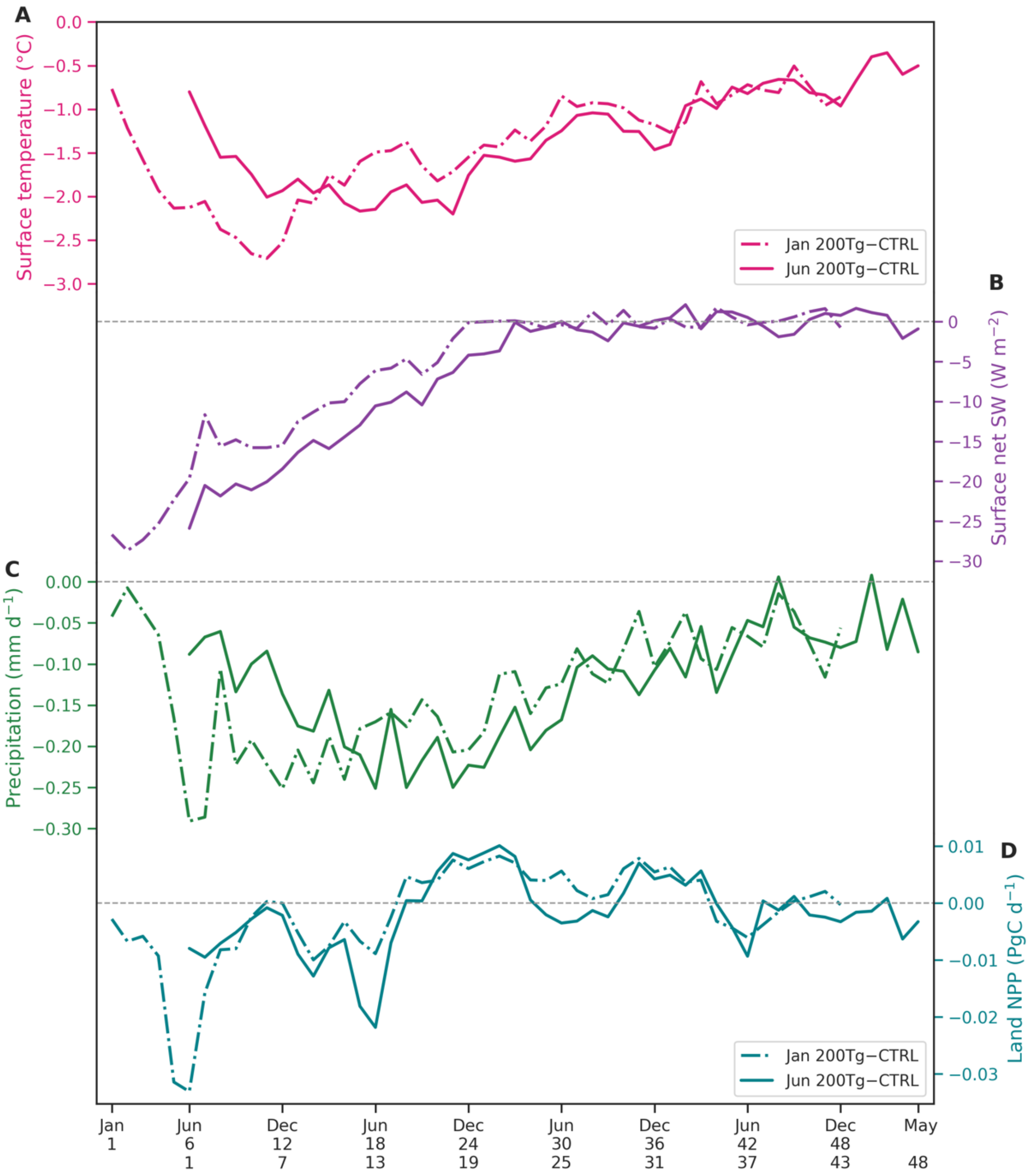

Fig. S19. Time series of globally averaged (**A**) surface temperature, (**B**) surface net shortwave flux, (**C**) precipitation, and globally integrated (**D**) land NPP anomalies for the two 200-Tg simulations with dust injections in January and June, respectively.

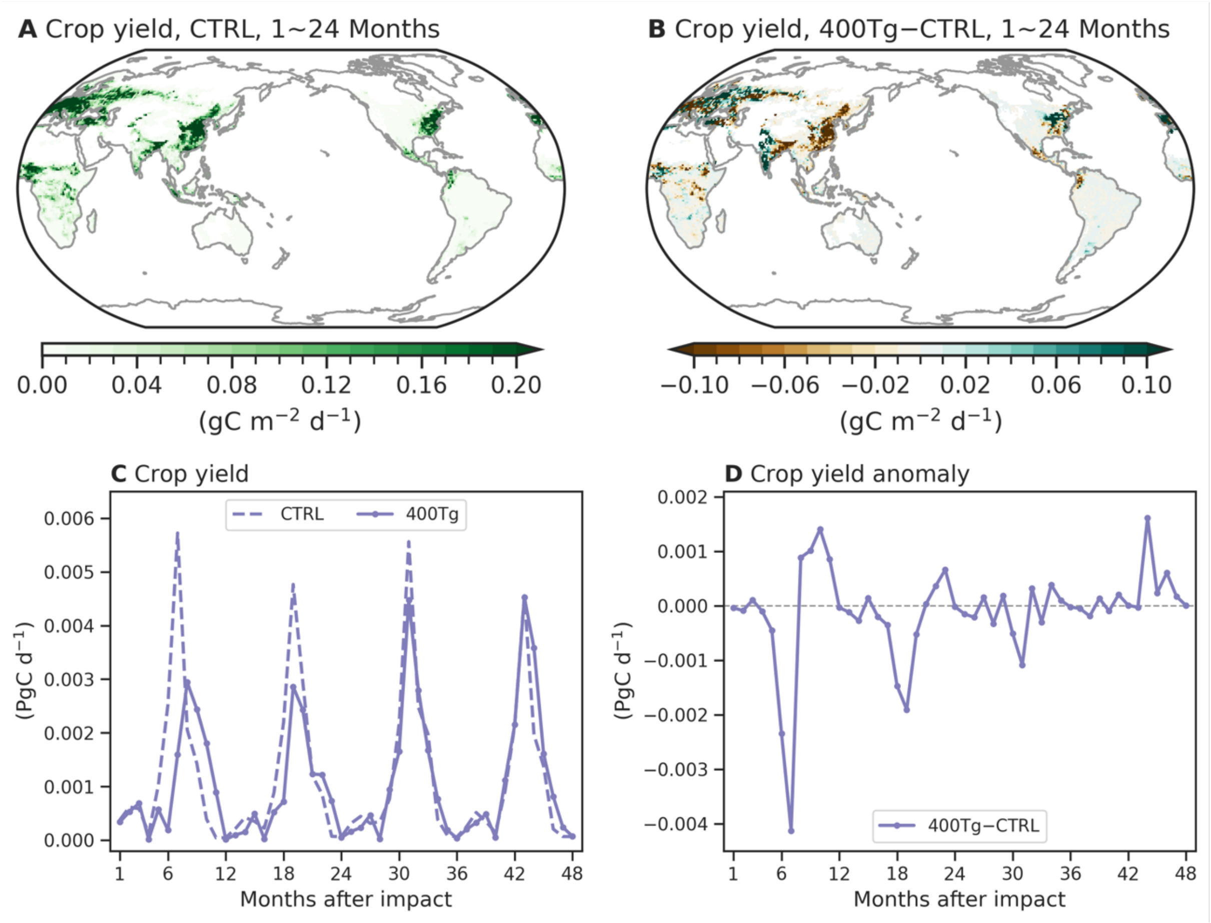

Fig. S20. Spatial patterns of crop yields (**A**) in the control simulation, (**B**) anomalies between the 400-Tg dust and control simulations averaged over 24 months after the impact. Time series of (**C**) globally integrated crop yields, and (**D**) the anomaly in the 400-Tg dust simulation for forty-eight months after the impact.

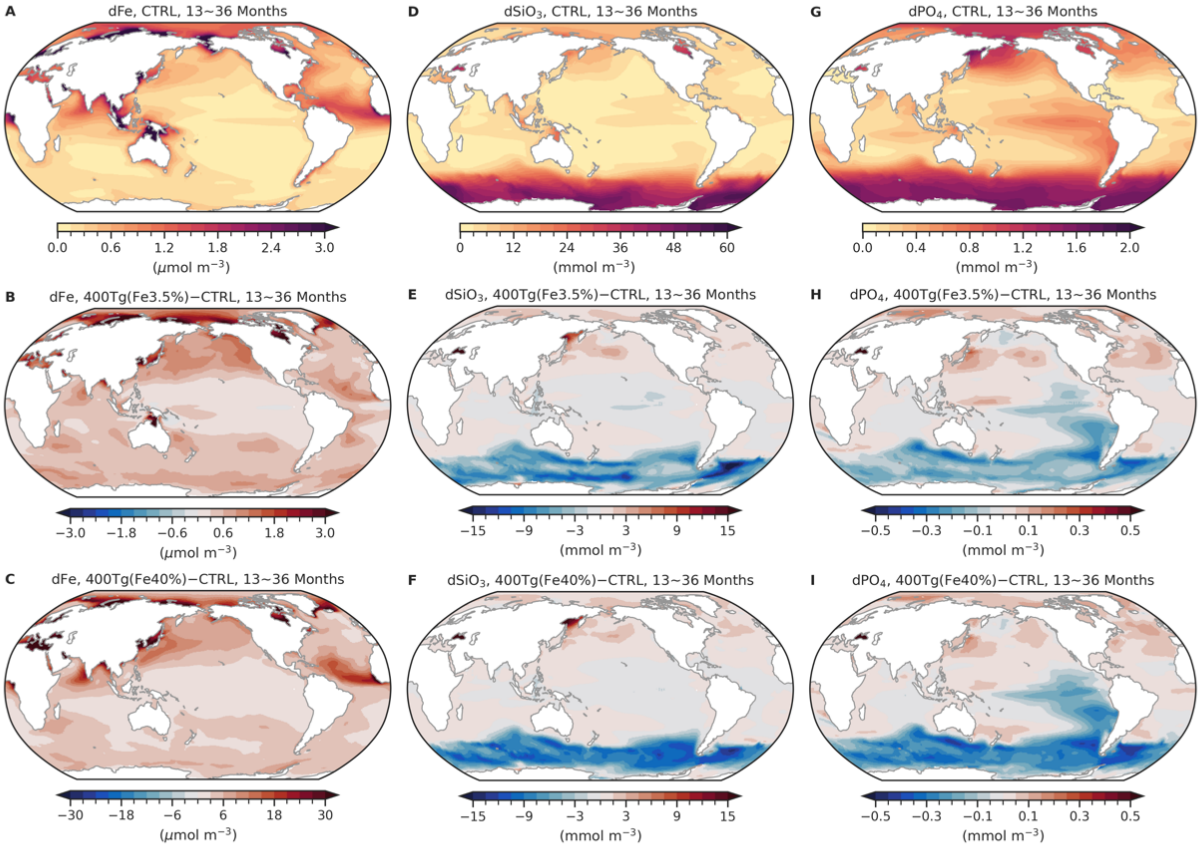

Fig. S21. Spatial patterns of (**A, B, C**) dissolved inorganic iron (Fe), (**D, E, F**) dissolved inorganic silicate (SiO_3), and (**G, H, I**) dissolved inorganic phosphate (PO_4) concentrations in the surface layer of 10 m averaged from 13 to 36 months after the impact. First (**A, D, G**), second (**B, E, H**) and third rows (**C, F, I**) represent the control simulation, and the anomalies of two 400-Tg dust simulations with 3.5% and 40% iron contents relative to the control simulation, respectively. Note the different units and color scales.

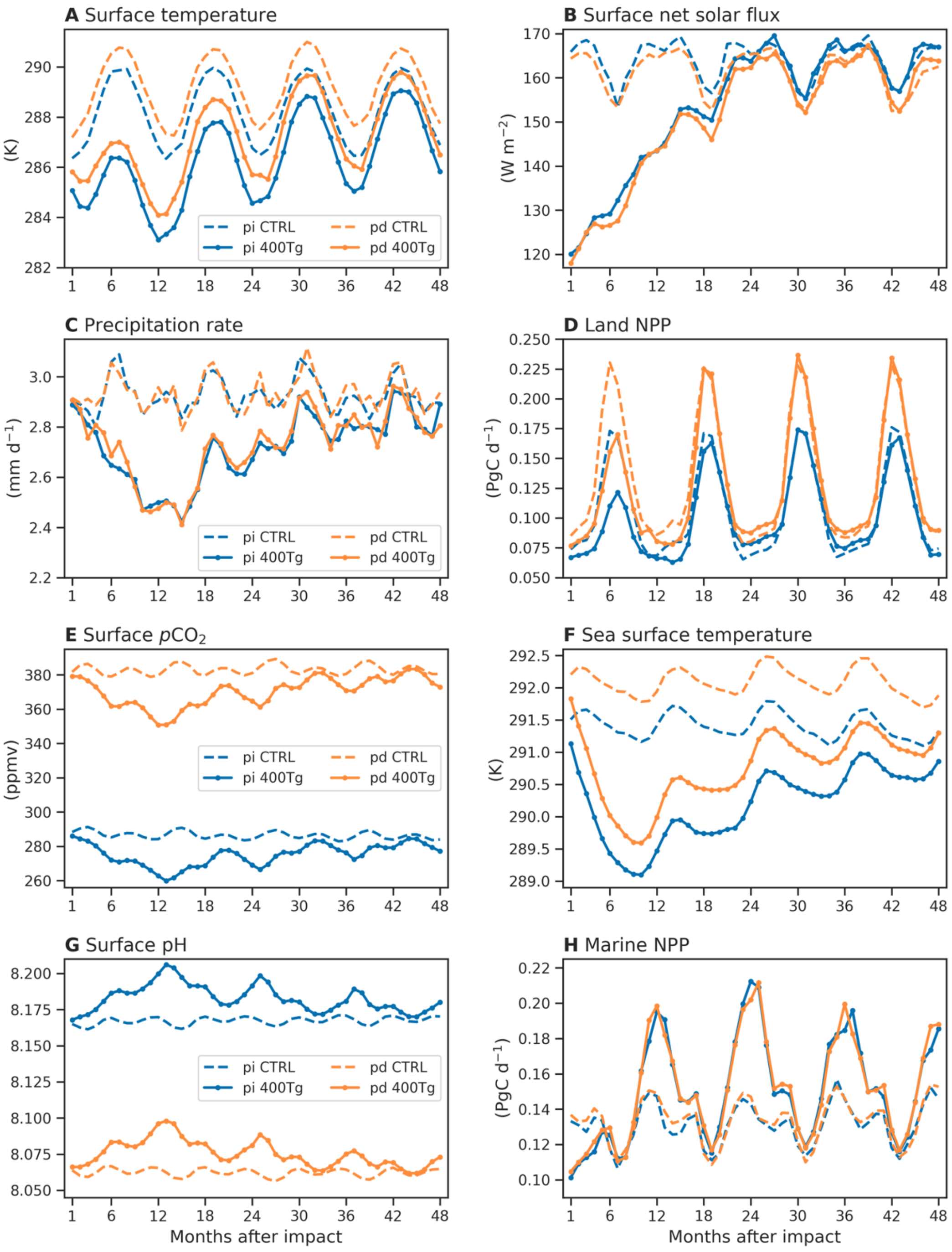

Fig. S22. Time series of globally averaged (**A**) surface temperature, (**B**) surface net shortwave flux, (**C**) total precipitation rate, (**E**) surface pCO_2, (**F**) sea surface temperature, (**G**) surface pH, and globally integrated (**D**) land and (**H**) marine NPP for forty-eight months after the impact. The control and the 400-Tg dust simulations are represented with dashed and solid lines, respectively. Blue and orange colors indicate the simulations under pre-industrial (pi) and present-day (pd) boundary conditions, respectively.

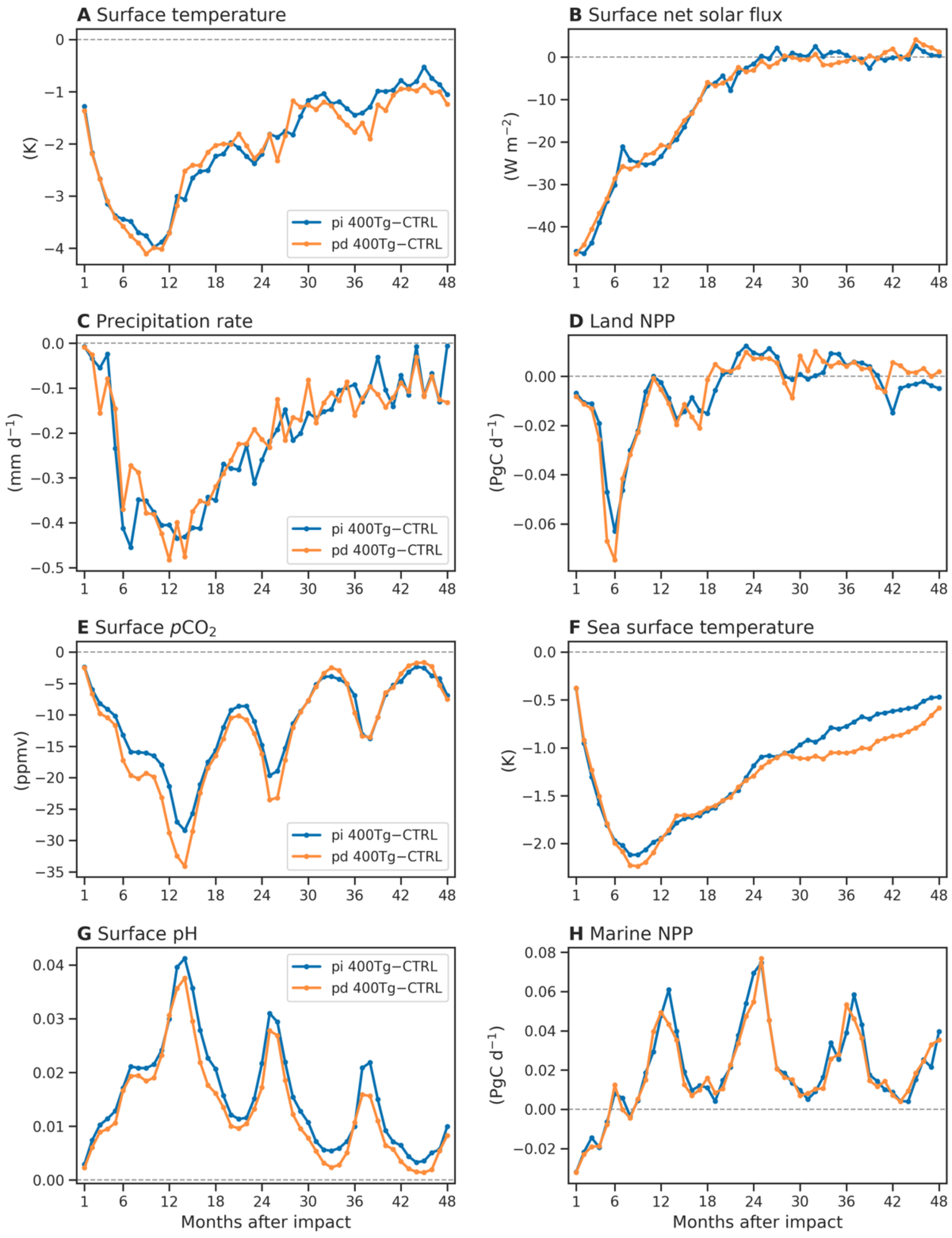

Fig. S23. As in Fig. S22, but for anomalies between the 400-Tg dust and control simulations.

Case name	Boundary condition	Injection amount	Injection time
Dust 400Tg	pre-industrial (pi)	400 Tg	January 1st
Dust 400Tg (Fe40%)	pi	400 Tg with 40% iron	January 1st
Dust 300Tg	pi	300 Tg	January 1st
Dust 200Tg	pi	200 Tg	January 1st
Dust 200Tg_June	pi	200 Tg	June 1st
Dust 100Tg	pi	100 Tg	January 1st
Control (CTRL)	pi	×	×
pd Dust 400Tg (Fe40%)	present-day (pd)	400 Tg with 40% iron	January 1st
pd Control (CTRL)	pd	×	×

Table S1. CESM2/WACCM6 simulations conducted in this study.
